# Chromosome‐Level Genomics and Historical Museum Collections Reveal New Insights Into the Population Structure and Chromosome Evolution of Waterbuck

**DOI:** 10.1111/mec.70218

**Published:** 2025-12-22

**Authors:** Corey Kirkland, Xi Wang, Carla Canedo‐Ribeiro, Lucía Álvarez‐González, David Weisz, Alexandria Mena, Judy St Leger, Olga Dudchenko, Erez Lieberman Aiden, Aurora Ruiz‐Herrera, Rasmus Heller, Tony King, Marta Farré

**Affiliations:** ^1^ School of Natural Sciences University of Kent Canterbury UK; ^2^ Department of Biology University of Copenhagen Copenhagen Denmark; ^3^ Departament de Biologia Cellular, Fisiologia i Immunologia Universitat Autònoma de Barcelona Cerdanyola del Vallès Spain; ^4^ Institut de Biotecnologia i Biomedicina Universitat Autònoma de Barcelona Cerdanyola del Vallès Spain; ^5^ The Center for Genome Architecture Baylor College of Medicine Houston USA; ^6^ SeaWorld San Diego San Diego California USA; ^7^ Cornell University College of Veterinary Medicine Ithaca New York USA; ^8^ The Center for Theoretical Biological Physics Rice University Houston Texas USA; ^9^ The Aspinall Foundation Port Lympne Reserve Kent UK; ^10^ Durrell Institute of Conservation and Ecology (DICE) University of Kent Canterbury UK

**Keywords:** chromosome evolution, genome assembly, historical DNA, population genomics, Robertsonian fusions

## Abstract

Advances in sequencing and chromosome‐scale assembly have brought non‐model animals into focus, deepening our understanding of genome and chromosome evolution. Here, we present the waterbuck ( *Kobus ellipsiprymnus* ) as an emerging model antelope for studying population dynamics and chromosome evolution. Waterbuck evolutionary history has been shaped by both climatic and geographic changes, as well as structural chromosome changes, principally Robertsonian (Rb) fusions. To provide new insights into waterbuck evolution, we generated a chromosome‐level genome assembly for the species using PacBio HiFi and Hi‐C sequencing. We further leveraged museum collections to perform whole genome sequencing (WGS) of 24 historical specimens. Combined with a previous WGS dataset (*n* = 119), this represents the largest study of waterbuck populations to date and reveals previously unrecognised population structure and gene flow between waterbuck populations, alongside several regions of high genomic differentiation between the two recognised subspecies. Notably, several differentiation hotspots occur near the centromeres of fixed and polymorphic Rb fusions, exhibiting signatures of low recombination and local population structure. These regions contain genes involved in development, fertility, and recombination. Our findings underscore the value of chromosome‐level genome assemblies, the critical role of historical collections in capturing fine‐scale population structure and gene flow in species with wide‐ranging distributions, and the potential evolutionary impacts of Rb fusions on genomic differentiation and recombination landscapes.

## 1 Introduction

The waterbuck ( *Kobus ellipsiprymnus* ) is a large antelope species composed of two subspecies, the common (*K. e. ellipsiprymnus*) and defassa (*K. e. defassa*) waterbuck, with their taxonomic classification based on rump fur colouration, molecular data and cytogenetics (Kingswood et al. 1998; Lorenzen et al. 2006; Wang et al. 2024). The species is distributed across central, eastern and southern Africa, with the common subspecies found east of the Great Rift Valley, from Somalia in the north to South Africa in the south, while the defassa subspecies is found to the west of the Great Rift Valley, covering a larger distribution from Guinea Bissau in West Africa to Kenya and Tanzania in East Africa, as well as populations in Angola and Zambia in the south.

The two subspecies have moderate levels of genetic and genomic differentiation caused by a separation across the Great Rift Valley (Lorenzen et al. 2006; Wang et al. 2024). Although this separation occurred during the mid‐Pleistocene, historical and recent gene flow has subsequently occurred across this geographic and climatic barrier (Wang et al. 2024). A hybrid zone exists in northern Tanzania and Kenya, where this admixture is suggested to be recent, with hybrid individuals reported with intermediate phenotypes (Lorenzen et al. 2006; Wang et al. 2024). Within each subspecies, population structure largely reflects geographic locality, with each subspecies further grouped into northern and southern population groups (Wang et al. 2024).

**FIGURE 1:**
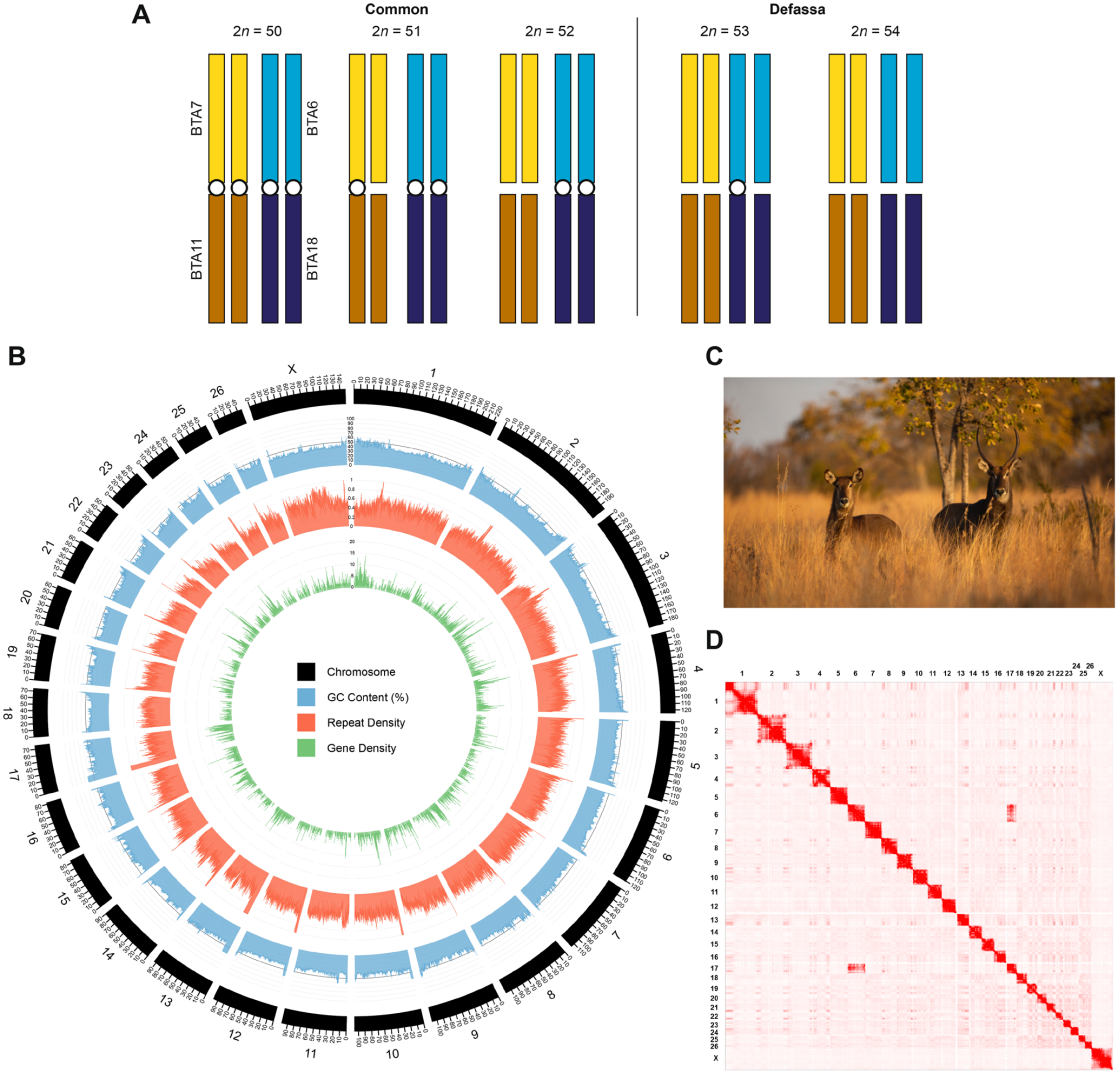
Chromosome‐level genome assembly generated for the defassa waterbuck (2*n* = 54). (A) Ideogram of the previously reported karyotypes in waterbuck (Kingswood et al. 1998) with the two polymorphic Robertsonian (Rb) fusions shown (homologous to cattle BTA6;18 and BTA7;11). Chromosomes are coloured by homology to cattle chromosomes and Rb fusions are depicted by a white circle. (B) Circos plot of waterbuck chromosomes, with GC content (%), repeat density, and gene density calculated in 100 Kb windows. (C) A female and male waterbuck. (D) Hi‐C interaction matrix for the 2*n* = 52 sample mapped to the chromosome‐level genome assembly (2*n* = 54), showing interchromosomal interactions between KEL6 and KEL17 (syntenic to cattle BTA6 and BTA18, respectively), as a result of the Rb fusion.

At the chromosome‐level, waterbuck show inter‐ and intra‐subspecies karyotypic variation in natural and captive populations due to the Robertsonian (Rb) fusion of two pairs of acrocentric chromosomes, syntenic to cattle ( *Bos taurus* ) chromosomes BTA6;18 and BTA7;11 (Kingswood et al. 1998; Kingswood et al. 2000) (Figure 1A). In the common waterbuck the BTA6;18 fusion is fixed, whilst the BTA7;11 fusion is polymorphic, resulting in three karyotypes: 2*n* = 52 (without the fusion), 2*n* = 51 (heterozygous for the fusion) or 2*n* = 50 (homozygous for the fusion). Whereas in the defassa, the BTA7;11 fusion is absent, and the BTA6;18 fusion has been found to be heterozygous (2*n* = 53) or absent (2*n* = 54) (Figure 1A). The remaining waterbuck karyotype is composed of three submetacentric autosomes, formed from historical Rb fusions that have become fixed within the species (syntenic to cattle chromosomes BTA1;19, BTA2;25 and BTA5;17), a metacentric X chromosome, an acrocentric or submetacentric Y chromosome, and 19–23 acrocentric autosomes (Kingswood et al. 1998; Kingswood et al. 2000).

Recent studies have investigated waterbuck population dynamics using genomic data (Wang et al. 2024), relying on a scaffold‐level short‐read assembly for waterbuck as the reference genome, alongside the chromosome‐level assembly of domestic goat ( *Capra hircus* ) (Chen et al. 2019). However, the available waterbuck reference genome consists of more than 88,000 scaffolds with an N50 of 0.78 Mb (Chen et al. 2019), limiting its utility for investigating large‐scale structural variation. Given that ruminant genomes are highly repetitive and characterised by multiple well‐documented chromosomal rearrangements (Arias‐Sardá et al. 2023)—and that waterbuck themselves exhibit karyotypic variation—a chromosome‐level genome assembly is essential for resolving these structural changes and understanding their evolutionary consequences. Moreover, the previous population genomic study (Wang et al. 2024) particularly focused on populations near the contact zone between the two subspecies. To reveal finer‐scale population structure across the species' broad African range, a more comprehensive and geographically diverse sampling of the species is needed.

Here, we sequenced and assembled a chromosome‐level genome for the waterbuck, achieving high contiguity and completeness. We then generated genomic sequences for 24 historical museum samples collected between 1900 and 1938 and integrated this with 119 previously published modern samples (Wang et al. 2024). Historical sampling enabled us to study population structure at both a broader and a more finely‐resolved scale than had previously been reported, uncovering novel admixture and gene flow in waterbuck. Additionally, using the newly generated high‐quality genome assembly, we investigated genomic differences at the chromosome‐level between the two subspecies of waterbuck for the first time, revealing putative signatures of Rb fusions in waterbuck and their potential impacts on chromosome evolution. Our study provides a greater understanding of the influence of population dynamics and chromosome rearrangements in the speciation process, particularly with large rearrangements such as fusions often understudied in wild species. We therefore provide a genomic framework for further work in waterbuck and other taxa.

## 2 Materials and Methods

### 2.1 Primary Mammalian Cell Culture and Karyotyping

A primary fibroblast mammalian cell line was established for a captive female defassa waterbuck ( *Kobus ellipsiprymnus defassa* ) from a tissue sample provided by the Aspinall Foundation (Kent, UK). The culture was maintained in DMEM containing 10% FBS and 1% Pen‐Strep, incubated at 37°C with 5% CO_2_. Cells were harvested on several passages and the stability of the karyotype was checked. Harvesting of chromosomes followed (Howe et al. 2014). Briefly, colcemid (10 μL/mL) was added to cell culture flasks and incubated at 37°C with 5% CO_2_ for 45 min. Cells were washed with 1× PBS. Trypsin was added to the flasks for 2 min before transferring contents to a tube. Tubes were centrifuged at 200 *g* for 5 min, followed by removal of the supernatant and resuspension in 5 mL of Carnoy's Fixative (3:1 methanol and glacial acetic acid) with vortexing. A further 5 mL of fixative was added without vortexing, centrifuged at 200 *g* for 5 min, and the supernatant discarded. This step was then repeated, and chromosome preps were stored at 4°C. Slides were stained with DAPI, and the metaphases were captured with fluorescent microscopy and karyotyped using SmartTypeDemo v3.3.2.

### 2.2 DNA Extraction and Long‐Read PacBio HiFi Sequencing

DNA was extracted from the primary cell line using the QIAGEN Blood and Cell DNA extraction kit following the cell culture protocol, including an RNase A treatment before lysis. DNA was eluted in 10 mM Tris–HCl (pH 8.5) and quantified using NanoDrop and Qubit. DNA was also measured for fragment lengths using pulse field gel electrophoresis (PFGE). DNA was sent to Edinburgh Genomics (Edinburgh, UK), where one PacBio SMRTbell library was prepared and sequenced on two PacBio Sequel IIe SMRT 8M Cells in HiFi mode.

### 2.3 De Novo Genome Assembly and Gene Annotation

The pipeline for genome assembly was adapted from the Vertebrate Genome Project (VGP) pipeline and is summarised below. HiFi reads in BAM format were converted to FASTQ format using Samtools v1.6 ‘fastq’ (Danecek et al. 2021), followed by quality control with FASTQC v0.11.9 (https://www.bioinformatics.babraham.ac.uk/projects/fastqc), MultiQC v1.0.dev0 (Ewels et al. 2016) and Nanoplot v1.32.1 (De Coster et al. 2018). Reads containing the adapters (ATCTCTCTCAACAACAACAACGGAGGAGGAGGAAAAGAGAGAGAT and ATCTCTCTCTTTTCCTCCTCCTCCGTTGTTGTTGTTGAGAGAGAT) were removed with Cutadapt v1.18 (Martin 2011) with parameters minimum error rate 0.1%, minimum overlap length 35, minimum length 5000 bp, maximum length 30,000 bp and discard trimmed reads. To estimate the genome size, the maximum read depth and the transition coverage between the haploid and diploid peaks we used a k‐mer‐based approach (32‐mer) with Meryl v1.3 (Rhie et al. 2020) and profiled using GenomeScope2 v2.0 (Ranallo‐Benavidez et al. 2020). The genome was assembled with Hifiasm v0.16.1‐r375 (Cheng et al. 2021), with purging of all types of haplotigs, a purge maximum of 45 and removal of 20 bp from both ends of the reads, which output a primary contig‐level genome assembly. Additionally, the mitochondrial genome was assembled with the MitoHiFi v2.2 pipeline (Uliano‐Silva et al. 2023). Genome statistics were produced with QUAST v5.0.2 (Mikheenko et al. 2018) and Merqury v1.3 (Rhie et al. 2020), and the genome was assessed for completeness and contamination with the BlobToolKit v2 pipeline (Challis et al. 2020) using BUSCO v5 (Manni, Berkeley, Seppey, and Zdobnov 2021; Manni, Berkeley, Seppey, Simão, and Zdobnov 2021), BLAST v2.10.0 and Uniprot 2023_01 (Consortium 2023) databases.

The Hi‐C library was prepared from the defassa waterbuck primary cell line and sequenced as described in (Álvarez‐González et al. 2022). Additional Hi‐C data was generated by DNA Zoo (https://www.dnazoo.org) following the protocol in (Rao et al. 2014) from a female common waterbuck blood sample bred in captivity, with a karyotype of 2*n* = 52. Both Hi‐C datasets were aligned to the contig‐level genome assembly with Juicer (Durand et al. 2016), however due to high levels of interchromosomal Hi‐C contacts in the defassa Hi‐C dataset, the common waterbuck Hi‐C dataset was used to scaffold the genome using 3D‐DNA (Dudchenko et al. 2017).

The chromosome‐level genome was reviewed and curated using Juicebox Assembly Tools (JBAT) (Dudchenko et al. 2018). The defassa Hi‐C dataset was used to resolve the karyotype to 2*n* = 54 and this was confirmed via synteny to the cattle reference genome ( *Bos taurus* ; ARS‐UCD2.0) and the previous cytogenetic study (Kingswood et al. 1998). The waterbuck genome was aligned to cattle with MashMap (Jain et al. 2018), syntenic blocks were constructed at 300 Kb resolution using several customised scripts, and then visualised with syntenyPlotteR v1.0.0 (Quigley et al. 2023) in R. Scaffolded chromosomes orthologous to BTA6 and BTA18 in the draft assembly were split into two chromosomes in the chromosome‐level waterbuck genome assembly. An interactive map was produced showing two Hi‐C datasets produced by DNA Zoo (2*n* = 52 and an additional unstudied karyotype; 2*n* = 51) aligned to the chromosome‐level genome assembly (https://www.dnazoo.org/post/don‐t‐go‐chasing‐waterbuck).

In this study, we further curated the genome assembly by reorientating chromosomes with their centromeres closer to the start and reordering by size (Table S1). Both sets of Hi‐C data (2*n* = 52 and 2*n* = 54) were then remapped to this final curated assembly, where the common Hi‐C data had strong interchromosomal interactions between waterbuck chromosomes KEL6 and KEL17, syntenic to BTA6 and BTA18, respectively (Figure 1D and Figure S3B), whilst the defassa Hi‐C did not have stronger interchromosomal interactions compared to other chromosomes (Figure S3C). The chromosome‐level genome assembly was annotated for repeats with RepeatMasker v2.6.0+ (http://www.repeatmasker.org) using the Dfam_Consensus‐20181026 and RepBase‐20181026 databases, and the *Bos taurus* dataset. Protein‐coding genes were annotated with GeMoMa v1.9 (Keilwagen et al. 2016, 2018) using both goat ( *Capra hircus* ; ARS1.2) and cattle ( *Bos taurus* ; ARS‐UCD2.0) as reference. Genes were filtered to exclude duplicate gene annotations with multiple transcripts or genes that had multiple annotations but the same coordinates.

### 2.4 Circos Plot

To visualise the final genome assembly and annotation, 100 Kb windows were created along each of the 27 chromosomes. The percentage of GC content was calculated with SeqKit v2.6.1 fx2tab (Shen et al. 2016). Repeat density was calculated as the total number of masked bases in each window divided by 100 Kb. Gene density was calculated as the number of protein‐coding genes in each 100 Kb window. Visualisation was performed using Circos 0.69.8 (Krzywinski et al. 2009).

### 2.5 Historical Museum Sampling and Sequencing

Historical waterbuck skin samples were provided by the Powell Cotton Museum (Kent, UK) and the Royal Museum for Central Africa (Tervuren, Belgium). DNA was extracted from skin samples using a modified phenol‐chloroform protocol, adapted from (McDonough et al. 2018; Roycroft et al. 2021) and Molecular Cloning Vol I, and described as follows. Skin samples were dissected into small sections (5–10 mm) using sterile equipment, briefly cleaned in a dilute bleach solution, cleaned with sterile ultrapure water. Samples were then incubated in water at room temperature, with frequent water changes, to clean and rehydrate the skin. Water was removed after 48 h and 320 μL lysis buffer (100 mM Tris–HCl pH 8.0, 5 mM EDTA pH 8.0, 200 mM NaCl and 0.2% SDS; Wang and Storm 2006), 40 μL Proteinase K, and 40 μL DTT were added to each tube. Samples were incubated at 56°C for up to 48 h and vortexed regularly. DNA was extracted using a standard phenol‐chloroform protocol with QIAGEN MaXtract tubes and eluted in TE buffer (pH 8.5). DNA was measured for quantity using NanoDrop and Qubit, and DNA length using gel electrophoresis. DNA extraction and quality control took place in a room dedicated to pre‐PCR work. A total of 24 samples were sent for Illumina library preparation (without size selection) and whole genome sequencing (WGS) at Novogene (UK), with a 2 × 150 bp read length and 350 bp insert size, on an Illumina NovaSeq6000.

### 2.6 Mapping of WGS Data and Filtering

Historical WGS data was quality controlled with FASTQC v0.11.9 and MultiQC v1.0.dev0 (Ewels et al. 2016) and the adapters (AGATCGGAAGAGCGTCGTGTAGGGAAAGAGTGTAGATCTCGGTGGTCGCCGTATCATT and GATCGGAAGAGCACACGTCTGAACTCCAGTCACGGATGACTATCTCGTATGCCGTCTTCTGCTTG) were trimmed and reads collapsed using AdapterRemoval v2.3.3 (Schubert et al. 2016) with parameters ‐‐mm 3, ‐‐collapse, ‐‐collapse‐conservatively, ‐‐trimns, and ‐‐trimqualities. Paired‐end and collapsed reads were aligned separately with BWA‐MEM v0.7.17 (Li 2013) to the assembled chromosome‐level waterbuck genome. BAM files were fixed and checked with Picard v3.0.0 FixMateInfomation and ValidateSAMFile (https://www.broadinstitute.github.io/picard/), and Samtools v1.6 calmd (Danecek et al. 2021). Duplicates were removed from paired‐end and collapsed BAM files with Picard v3.0.0 MarkDuplicates and the PALEOMIX v1.3.7 rmdup_collapsed script (Schubert et al. 2014), respectively. DNA damage was assessed, and quality scores were rescaled based on damage, using mapDamage v2.2.0 (Jónsson et al. 2013), with no filtering threshold set for samples. Paired‐end and collapsed BAM file flags were modified with Samtools v1.6 view and merged with Samtools v1.6 merge (Danecek et al. 2021). Mapping statistics for the merged alignment files were calculated with Samtools v1.6 stats and depth (Danecek et al. 2021) and Qualimap v2.2.2‐dev (Okonechnikov et al. 2016). Moreover, published WGS reads from 119 modern waterbuck samples from 10 populations (Wang et al. 2024) was mapped to the chromosome‐level reference genome using a modified version of the PALEOMIX bam pipeline (Schubert et al. 2014). Lastly, BAM files were filtered for secondary and supplementary alignments, PCR duplicates, quality, insert sizes < 50 bp or greater than 1000 bp, reads with < 50 bp or 50% mapped, and reads mapping to different scaffolds or with unexpected orientations.

Genomic sites were then filtered in both the reference genome and the alignment files as described in (Wang et al. 2024). Briefly, genomic sites on unplaced scaffolds and sex chromosomes, and sites annotated as repetitive, were firstly removed. Then regions with excess heterozygosity were identified in the 24 historical BAM files and removed. The historical BAM files were also used to calculate the global depth across WGS samples and remove sites below the 1st percentile and above the 99th percentile. Finally, sites with low mappability were excluded. This resulted in a filtered genomic sites file which was used when calling genotype likelihoods.

### 2.7 Heterozygosity and Effective Population Size

To calculate overall genome‐wide heterozygosity per sample for filtered sites, ANGSD v0.940 (Korneliussen et al. 2014) was used (‐doSaf 1, ‐GL 2, ‐minMapQ 30 and ‐minQ 20) to produce site allele frequency (SAF) files, with the reference genome used as the ancestral state. Transversions were also removed (‐noTrans 1) to consider differences between all sites and transversion‐specific sites. SAF files were then converted into the folded site frequency spectrum (SFS) files by the ANGSD v0.940 realSFS program (Korneliussen et al. 2014) and plotted in R.

The effective population size (Ne) was calculated for three modern waterbuck populations (Samole, QENP and Matetsi) with greater than 10 individuals and no previously reported admixture (Wang et al. 2024) using GONE2 (Santiago et al. 2025). BCF files for each population were created with ANGSD v0.940 (Korneliussen et al. 2014) on filtered sites (‐gl 1, ‐dopost 1, ‐domajorminor 1, ‐domaf 1, ‐dobcf 1, ‐dogeno 1, ‐docounts 1, ‐SNP‐pval 1e‐6, ‐minQ 20, ‐minMapQ 30). BCF files were converted to VCF with bcftools v1.22 (Danecek et al. 2021) and inputted into Plink v1.9 (Purcell et al. 2007) to recode into PED and MAP files (‐‐recode, ‐‐maf 0.05, ‐‐allow‐extra‐chromosome). PED and MAP files were then used in GONE2 v1.0.2 (Santiago et al. 2025) with genotyping data set to low coverage (‐g 3), average base calling error rate per site of 0.001 (‐b 0.01), and a constant recombination rate of 1 cM/Mb (‐r 1). A generation time of 7 years was used to transform generations into the number of years before sampling.

### 2.8 Population Structure

Genotypes likelihoods were called per chromosome on filtered sites with ANGSD v0.940 (Korneliussen et al. 2014) (‐GL 2, ‐doMajorMinor 1, ‐doMaf 1, ‐SNP_pval 1e‐6, ‐minMaf 0.05, ‐minMapQ 30 and ‐minQ 20). Genotypes were also calculated for only transversion sites (‐rmtrans 1). Beagle likelihood files were combined for all chromosomes and samples, and the combined beagle file was used in both the principal component analyses (PCA) and admixture analyses. PCAs were constructed using pcangsd v.0.99 (Meisner and Albrechtsen 2018) with ‐minMaf 0.05, whilst admixture proportions were calculated with ANGSD v0.940 NGSadmix (Skotte et al. 2013) with ‐minMaf 0.05 and estimated populations of K between K = 2 and K = 20, with K = 2, K = 3, K = 4 and K = 12 plotted in this study using R as they were the most informative. NJ trees were constructed with ANGSD v0.940 (Korneliussen et al. 2014) (‐GL 2, ‐minMapQ 30, ‐minQ 20, ‐doMajorMinor 1, ‐doMaf 1, ‐SNP_pval 1e‐6, ‐doIBS 1, ‐doCounts 1, ‐doCov 1, ‐makeMatrix 1 and ‐minMaf 0.05). The NJ tree was constructed using the pairwise IBS matrix with a custom R script.

Estimated Effective Migration Surfaces (EEMS) were calculated by firstly running ANGSD v0.940 (Korneliussen et al. 2014) using parameters ‐GL 2, ‐minMapQ 30, ‐minQ 20, ‐doMajorMinor 1, ‐doMaf 1, ‐SNP_pval 1e‐6, ‐doIBS 1, ‐doCounts 1, ‐doCov 1, ‐makeMatrix 1 and ‐minMaf 0.05. The EEMS runeems_snps pipeline (Petkova et al. 2015) was run in triplicates (nIndiv 143, nSites 143, nDemes 600, diploid, numMCMCIter 20000000, numBurnIter 10000000 and numThinIter 9999) and visualised with the library rEEMSplots in R.

### 2.9 Genomic Differentiation and Detecting Signatures of Putative Chromosome Rearrangements

Genomic differentiation (F_ST_) was estimated between the two subspecies of waterbuck, *K. e. ellipsiprymnus* (common) and *K. e. defassa* (defassa), by running ANGSD v0.940 (Korneliussen et al. 2014) separately for the two subspecies on filtered sites (‐GL 2, ‐doSaf 1, ‐minMapQ 30 and ‐minQ 20) generating unfolded SAF files. Folded SFS files were generated with ANGSD v0.940 realSFS (Korneliussen et al. 2014) with parameter ‐fold 1, followed by fst index (‐whichFst 1 and ‐fold) and fst stats2 (window size and step size of 10 Kb). To find genes within the F_ST_ windows across the genome, the output file was intersected with the waterbuck homology‐based gene annotation file using BEDTools intersect v2.31.0 (Quinlan and Hall 2010). Plots were created from F_ST_ values with the addition of synteny data to cattle chromosomes using a custom R script.

LD was calculated using several methods. Genotype likelihoods were computed for each chromosome on filtered genomic sites with ANGSD v0.940 (Korneliussen et al. 2014) using parameters ‐minMapQ 30, ‐minQ 20, ‐doCounts 1, ‐GL 2, ‐doMajorMinor 1, ‐doMaf 1, ‐skipTriallelic 1 ‐doGlf, ‐SNP_pval 1e‐6, ‐remove_bads, ‐minMaf 0.05. The program ngsLD v1.2.1 (Fox et al. 2019) was used with parameters ‐‐max_kb_dist 1000 and ‐‐rnd_sample 0.1. Mean LD was then calculated in 100 Kb windows across chromosomes using a custom R script. Mean pairwise LD was also calculated in 1 Mb windows on chromosomes involved in putative rearrangements using a custom R script. We also calculated interchromosomal LD between KEL6 and KEL17 by computing genotype likelihoods on the two chromosomes and calculating LD using ngsLD (Fox et al. 2019) with the parameters ‐‐max_kb_distance 0 and ‐‐rnd_sample 0.001. Files were filtered for interchromosomal interactions and mean pairwise LD was calculated in 1 Mb windows.

Local PCAs of particular regions of interest were computed by filtering the genomic sites file and genotyping with ANGSD v0.940 (Korneliussen et al. 2014). The PCAs were then computed on the 143 individuals and visualised as described previously.

Gene ontology (GO) statistical overrepresentation tests were computed with Panther (Mi et al. 2019; Thomas et al. 2022) with *Bos taurus* as reference on lists of waterbuck genes found within the 99th percentile of F_ST_ windows or in blocks of high F_ST_.

## 3 Results

### 3.1 Sequencing and Assembling a Chromosome‐Level Genome for the Waterbuck

In order to explore population structure and chromosome evolution within the waterbuck at the chromosome level, a high‐quality genome was required. A newly established cell culture of a female defassa waterbuck was karyotyped, showing a standard karyotype for the defassa of 2*n* = 54, with no evidence of the two polymorphic Rb fusions (Figure S1). The sample was then sequenced with PacBio HiFi (21× coverage, read N50: 18.89 Kb) and assembled to contig‐level (3.15 Gb, 1071 contigs, N50: 71.17 Mb, L50: 17; Figure S2A).

To scaffold the genome to chromosome‐level, Hi‐C data was generated and sequenced from the defassa sample (2*n* = 54, XX) and an additional female common waterbuck sample (2*n* = 52, XX; Figure S3A,B). Due to better data quality, Hi‐C scaffolding was performed using data from the common waterbuck sample. Hi‐C data from the defassa waterbuck sample was subsequently used to help resolve the genome assembly to match the defassa subspecies (2*n* = 54 karyotype), and this was confirmed with synteny to cattle chromosomes (Figure S3A,D) and the previous cytogenetic study (Kingswood et al. 1998). We then further curated the genome by reorienting and reordering chromosomes by size (Table S1). This resulted in a final curated genome assembly for the defassa waterbuck (Figure 1B,D, Figures S2B and S3). The chromosome‐level genome was highly contiguous (3.15 Gb, 1014 scaffolds, N50: 98.45 Mb, and L50: 12) containing 26 autosomes and one sex chromosome (Figure 1D). This improved the N50 of the previous scaffold‐level assembly (Chen et al. 2019) (0.78 Mb) by 125 times. The final assembly presents 96.27% of BUSCO mammalian genes, with 94.41% being single copy (Figure S2B). The mitochondrial genome was also assembled from the PacBio HiFi reads, generating a genome of 16,427 bp in length including 37 genes, of which 13 were protein coding, 22 were tRNA, and two were rRNA (Figure S4).

A total of 24,645 protein‐coding genes were predicted using homology‐based annotation with cattle ( *Bos taurus* ; ARS‐UCD2.0) and goat ( *Capra hircus* ; ARS1.2) as references (Figure 1B and Table S2). Around 54.80% (1.73 Gb) of the genome was repetitive, with LINEs representing the majority of repeats (24.26%), followed by satellite or centromeric repeats (14.24%), and SINEs (9.70%; Table S3). GC content (%) was highest at the start and ends of chromosomes, while repeat density was highest in regions surrounding the expected locations of centromeres (Figure 1B).

### 3.2 A Combined WGS Dataset of Historical and Modern Waterbuck Samples

**FIGURE 2:**
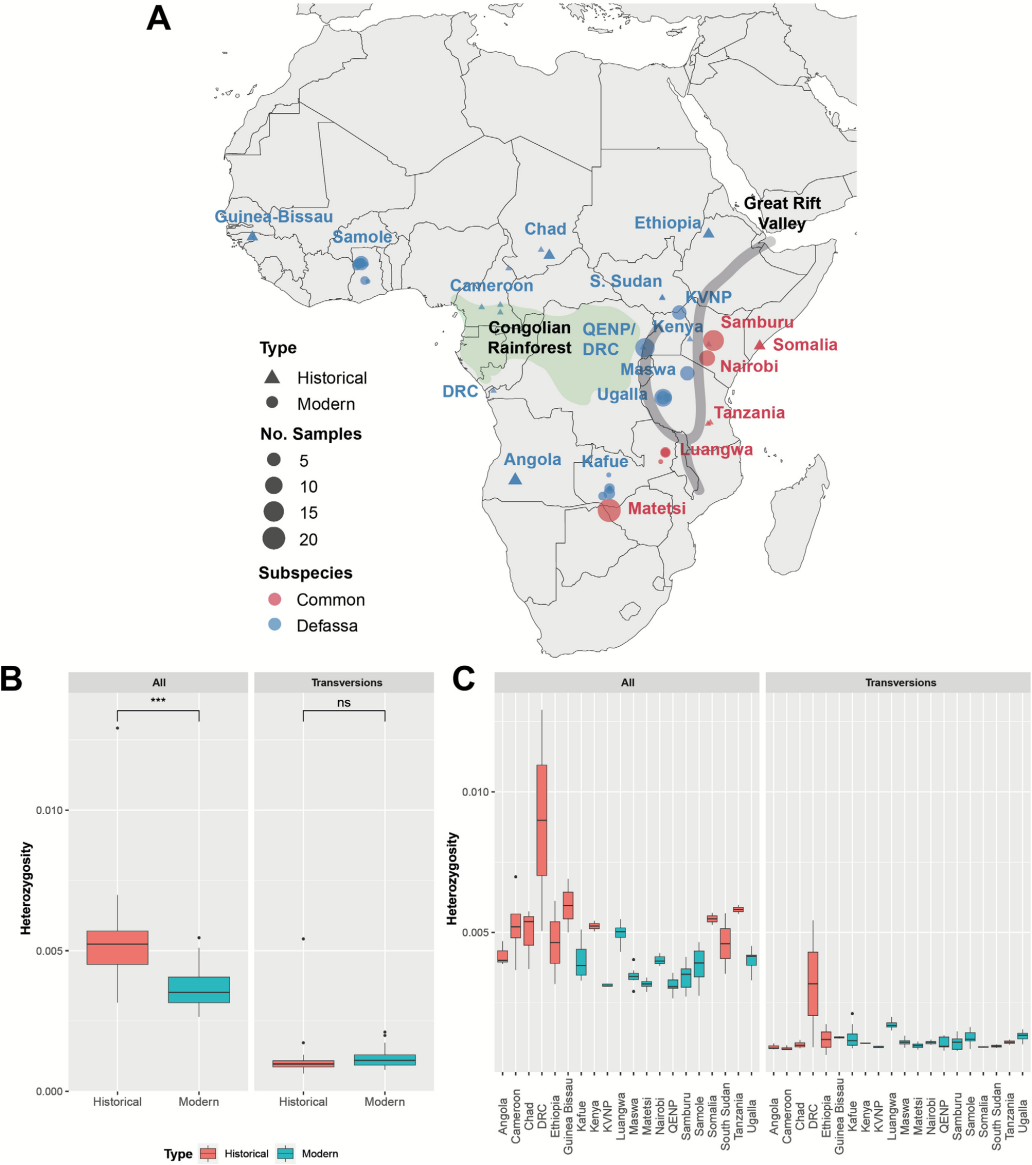
Historical and modern sampling of waterbuck. (A) Locations of the historical and modern samples, with the approximate position of the Congolian Rainforest and the Great Rift Valley annotated. (B) Genome‐wide heterozygosity grouped by age. (C) Genome‐wide heterozygosity grouped by population. Heterozygosity was calculated on all genomic sites or only transversion sites.

To provide a wider sampling distribution across the species range we utilised two museum collections. In total we sampled 24 historical waterbuck skins (dating between 1900 and 1938), extracted historical DNA (hDNA), and performed WGS at low coverage (~5×; Figure 2A and Table S4). Of the historical samples, 20 were taxonomically classified as the defassa subspecies (from locations in Angola, Cameroon, Chad, Democratic Republic of Congo; DRC, Guinea Bissau, South Sudan and Tanzania) and four as common (from locations in Ethiopia and Somalia). We integrated these historical genomes with 119 modern WGS data from a previous publication (Wang et al. 2024) (Figure 2A and Table S4). Modern data included six populations classified as defassa from Uganda (QENP and KVNP), Zambia (Kafue), Tanzania (Maswa and Ugalla) and Ghana (Samole), and four common waterbuck populations from Zimbabwe (Matetsi), Zambia (Luangwa) and Kenya (Samburu and Nairobi).

All WGS data was mapped to the waterbuck chromosome‐level genome assembly, with an average coverage of 4.03× across the 143 samples (Table S4). To produce a set of high confidence SNPs and genomic areas for downstream analyses, we conservatively filtered genomic sites, by removing repetitive regions, positions with excess heterozygosity, low mappability and regions with low or high depth of coverage. This resulted in 40.19% of the reference genome available for our analyses (Table S5). As historical samples are prone to DNA damage over time, we also estimated DNA damage of the mapped sequencing reads for the 24 historical samples (Figure S5). C to T substitutions were highest at the starts of paired end reads, with sample ‘WB_1b_5X’ having the highest rate (0.026) and sample ‘WB_2h_5X’ the lowest (0.006). Whereas G to A substitutions were highest at the ends of collapsed reads (0.090 to 0.044). However, we deemed this level of DNA damage to be acceptable for our analyses and mapping quality scores were subsequently rescaled.

We then calculated genome‐wide heterozygosity on all genomic sites for the historical and modern samples and compared this to transversion sites (Figure 2B). Historical samples had higher heterozygosity on average than modern samples when using all sites (*p* = 0.0001), but no significant difference when using only transversion sites (*p* = 0.8271), suggesting there may be some differences due to historical DNA damage, rather than a change in heterozygosity due to any population decline. The DRC sample ‘WB_3k_5X’ had extremely high levels of heterozygosity (0.0129 for all sites and 0.0054 for transversion sites; Figure 2) and therefore was removed from the EEMS and F_ST_ analyses. Focusing on transversion sites for historical and modern populations, Luangwa had the highest average heterozygosity of all populations (0.0017), whereas Angola, Cameroon and KVNP had the lowest average heterozygosity (0.0009; Figure 2C).

Additionally, we calculated the effective population size (Ne) of three modern populations of waterbuck that had more than 10 individuals and with no previously detected admixture between subspecies (Wang et al. 2024). The defassa populations of Samole and QENP had stable Ne over the last 700 years, whereas the common population of Matetsi had a steep decline followed by stable Ne over the last 400 years (Figure S6). Together with the heterozygosity results, this suggests that waterbuck populations have remained relatively stable between historical and modern sampling periods. We therefore combined the 23 (or 24) historical genomes from the 10 newly sampled populations with 119 modern genomes from 10 previously published populations (Wang et al. 2024) to investigate recent population structure and dynamics.

### 3.3 Population Dynamics Across Waterbuck Range

**FIGURE 3:**
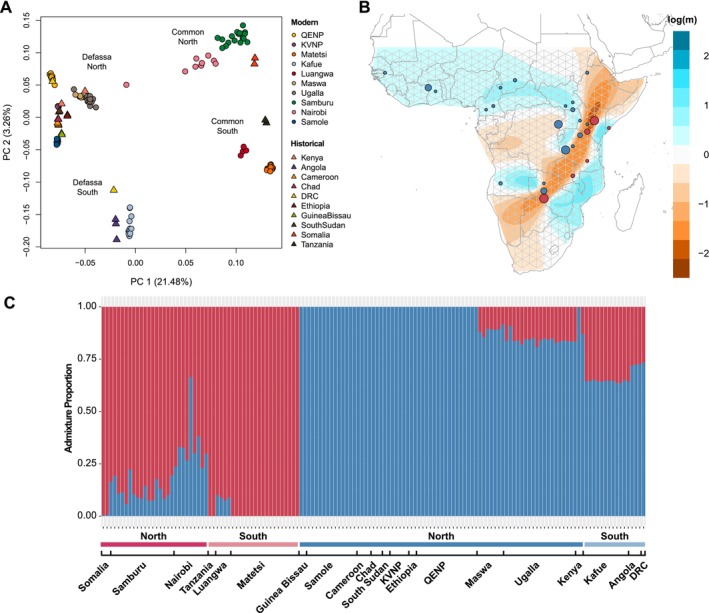
Fine‐scale population structure and gene flow in waterbuck. (A) PCA using all genomic sites grouped by population. (B) EEMS log migration rates (*m*) between populations, with common populations indicated by red circles and defassa blue circles. The size of the circles represents the number of samples at a given location. (C) Admixture proportions at K = 2 grouped by populations, subspecies, and geographical location. Red indicates common waterbuck and blue defassa waterbuck.

To explore population structure across the waterbuck's distribution, principal component analyses (PCA), admixture proportions (at K = 2, K = 3, K = 4 and K = 12 estimated populations), and overall F_ST_ were computed on genomic sites across the 26 autosomal chromosomes. Overall, PC1 (21.48%) of the PCA showed a split between the two subspecies, with the exception of one sample from Nairobi, which had previously been shown to be recently admixed (Wang et al. 2024), while PC2 (3.26%) grouped populations in the north and south separately (Figure 3A). A similar PCA result was found using only transversion sites, confirming that historical samples were not causing differences in genomic variability due to DNA damage (Figure S7). Population structure was also supported by moderate genomic differentiation (F_ST_ = 0.214) between the two subspecies; and between northern and southern groups in the common and defassa populations (F_ST_ = 0.115 and 0.139, respectively; Figure S8).

Admixture analysis at K = 2 grouped individuals into the two subspecies, with varying proportions of admixture found in samples closest to the contact zone (Samburu, Nairobi, Luangwa, Maswa, Ugalla and Kenya) and surprisingly in all samples in the Defassa South group (Kafue, Angola and DRC) (Figure 3C). At K = 3 defassa were split into two populations and common into one, at K = 4 defassa were split into three populations and common into one, and at K = 12 individuals were grouped more similarly to their geographic locality and populations (Figure S9).

Estimated Effective Migration Surface (EEMS) was used to visualise gene flow and genetic diversity between populations (Figure 3B and Figure S10). A strong barrier to gene flow was found across the subspecies divide, reflecting the location of the Great Rift Valley. Decreased gene flow was also found around the Congolian Rainforest, creating an historical barrier between northern and southern defassa populations, which extends towards the Great Rift Valley (Figure 3B). Mean diversity rates were highest between populations in East Africa around the Great Rift Valley (Ugalla, Maswa, Nairobi, Samburu, Somalia and Luangwa), whilst lowest in the northwest (Guinea Bissau and Samole) and Ethiopia (Figure S10). These results suggest that population structure in waterbuck has been shaped by climatic and geographical barriers, but with historical and ongoing gene flow near contact zones.

### 3.4 Tracing Genomic Signatures of Divergence Between Subspecies

**FIGURE 4:**
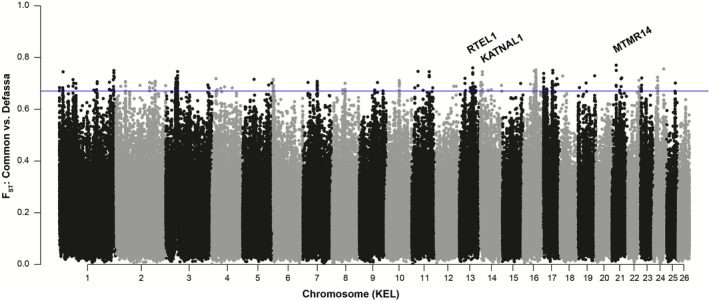
Genomic differentiation (F_ST_) calculated in 10 Kb windows between the common and defassa subspecies across waterbuck chromosomes (KEL). The blue line represents the 99th percentile of F_ST_ windows.

Given that waterbuck showed substantial population structure between the two subspecies (Figure 3A), we investigated which regions of the genome were highly differentiated, using our newly assembled chromosome‐level reference genome to place this for the first time into the context of waterbuck chromosomes (KEL). F_ST_ was estimated in 10 Kb windows containing greater than 1000 sites after filtering (Figure 4). We found 242 windows with F_ST_ ≥ 0.671, representing the 99.9th percentile. Overlapping these windows were 104 protein‐coding genes (Table S6), with the highest F_ST_ windows containing genes such as MTMR14 (KEL21; F_ST_ = 0.770) and RTEL1 (KEL13; F_ST_ = 0.760). Moreover, additional regions with high levels of genomic differentiation contained genes related to chromatin (KMT2C, PPP1CA/PPP1CB and BPTF), microtubules (STMN2, KATNAL1, KATNA1 and RABGAP1), and embryogenesis (LEUTX and HELZ).

When analysing the Gene Ontology (GO) terms of the 104 genes located in highly differentiated regions, we found these genes were statistically overrepresented for five Biological Process GO terms (negative regulation of t‐circle formation, linoleic acid metabolic process, hepoxilin biosynthetic process and lipoxygenase pathway) and three Molecular Function GO terms (RNA polymerase II binding, phosphatidylinositol activity and linoleate activity; Table S7). These results provide new insights into the impact of genomic differentiation on genic regions of the genome.

### 3.5 Putative Signatures of Chromosome Rearrangements

Using our new chromosome‐level genome, we next explored the chromosomes involved in fixed (KEL1, KEL2 and KEL3) and polymorphic (KEL6;17 and KEL8;9) Rb fusions in more detail. We identified regions of high F_ST_ between the two subspecies near the centromeres of chromosomes involved in fixed and polymorphic Rb fusions within waterbuck, with centromeres defined based on known karyotypes in the species (known acrocentric and submetacentric chromosomes), synteny and regions of higher repeat density (Figure 1).

**FIGURE 5:**
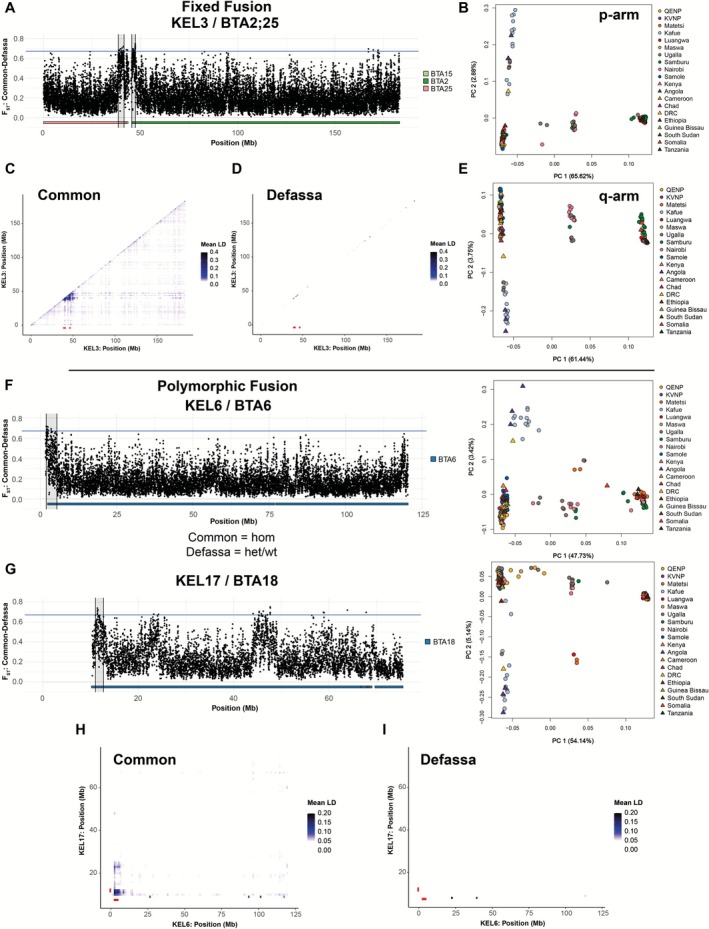
Signatures of Robertsonian (Rb) fusions in waterbuck. (A) F_ST_ (calculated in 10 Kb windows) of a fixed Rb fusion in KEL3 homologous to cattle BTA2;25. The region of interest was highlighted in grey and homology to cattle chromosomes was plotted below. (B) PCA of the region on the p‐arm of KEL3. PCAs were computed for each region using all 143 samples. (C) Mean pairwise LD across KEL3 in common and (D) defassa. Pairwise LD (calculated in 1 Mb windows) for each subspecies with regions of interest shown in red. (E) PCA of the region on the q‐arm of KEL3. (F) F_ST_ of KEL6 (BTA6) involved in the KEL6;17 (BTA6;18) polymorphic Rb fusion and PCA of the highlighted region. (G) F_ST_ of KEL17 (BTA18) and PCA of the highlighted region. (H) Mean pairwise interchromosomal LD between KEL6 and KEL17 in common and (I) defassa waterbuck.

Notably, we identified two blocks of high F_ST_ in the pericentromeric region of KEL3, a fixed Rb fusion in waterbuck (and fixed in the genus *Kobus*) (Kingswood et al. 2000), homologous to cattle chromosomes BTA2 and BTA25 (Figure 5A). These blocks, spanning 38.425–41.405 Mb and 45.415–47.265 Mb on KEL3, had elevated F_ST_ compared to adjacent genomic regions (99.9th percentile of the F_ST_ windows in the region was 0.719 and 0.744, respectively). In the common waterbuck, these regions showed elevated linkage disequilibrium (LD) across the centromere, suggesting that recombination is suppressed in this entire region in this subspecies (Figure 5C,D, and Figure S11). In contrast, KEL1 and KEL2—also fixed Rb fusions—did not display similar patterns of F_ST_ or LD (Figure S12).

Local PCAs of each of the two regions on KEL3 revealed three distinct clusters along PC1, explaining 61.440% and 65.620% of the variance, respectively (Figure 5B,D). This structure differed from the genome‐wide PCA (Figure 3A), suggesting a potential signature of the Rb fusion or a novel chromosomal rearrangement between subspecies. In these local PCAs, defassa populations clustered on the left (and one common Nairobi sample), common populations on the right (and two defassa Ugalla samples), and individuals from populations of both subspecies from the contact zone in the centre (defassa: Ugalla, common: Nairobi and Samburu).

Within these genomic regions, we identified a total of 128 genes (Table S6). The region on the p‐arm of KEL3 contained several genes involved in male fertility, including the genes IFT140 (F_ST_ = 0.720), PRSS21 (F_ST_ = 0.643) and SEP12 (F_ST_ = 0.621). The region of high F_ST_ on the q‐arm contained 15 genes and included OCA2 (F_ST_ = 0.626), part of the mammalian pigmentary system. Genes in these regions were statistically overrepresented for the GO term pre‐synapse (Table S7).

We then examined the chromosomes involved in the two polymorphic Rb fusions in waterbuck, to investigate the relationship between the fusion event and genomic differentiation and recombination. We found high genomic differentiation (F_ST_) near the centromeres of waterbuck chromosomes KEL6 (BTA6) and KEL17 (BTA18), involved in the KEL6;17 (BTA6;18) Rb fusion (Figure 5F,G), with blocks defined between 1.955–5.065 Mb on KEL6 (F_ST_ 99.9th percentile: 0.716) and 11.035–12.865 Mb on KEL17 (F_ST_ 99.9th percentile: 0.735). These regions also had higher intrachromosomal LD in the common subspecies than in the defassa (Figure S11). We then calculated interchromosomal LD between KEL6 and KEL17 for each subspecies and overall was higher in the common waterbuck than the defassa (Figure 5H,I). The highest interchromosomal LD was found between regions at the starts of both chromosomes, near the centromeres where the two chromosomes fuse. The higher interchromosomal LD across the two chromosomes in the common subspecies was expected, as the KEL6;17 Rb fusion is fixed in all karyotypes (2*n* = 50–52) and therefore is a large metacentric Rb chromosome. Whereas in the defassa the Rb fusion is either in the heterozygous form (2*n* = 53) or completely absent (2*n* = 54; Figure 1A) (Kingswood et al. 1998), and therefore interchromosomal LD would be much lower. We did not find the same signatures for the other polymorphic Rb fusion in waterbuck, KEL8;9 (BTA7;11; Figure S13). However, this polymorphic Rb fusion is absent in defassa waterbuck (2*n* = 53/54) and only present in the 2*n* = 50/51 karyotype in common.

PCAs of the two regions near the centromeres of KEL6 and KEL17 resulted in different population structure, with three groupings on PC1 (with a variance of 47.73% and 54.1.4%; Figure 5F,G), supporting a potential polymorphic variation. The left group contained individuals from defassa waterbuck populations (except the highly admixed individual from the Nairobi population; Figure 3C), the right group contained exclusively individuals of the common waterbuck populations, while individuals in the centre group belonged to populations along the contact zone for both subspecies (common: Luangwa, Matetsi, Nairobi and Samburu and defassa: Ugalla).

On KEL6 a total of 16 annotated genes were found in the region of interest, whilst 42 genes were found on KEL17 (Table S6). These two regions included several genes such as APELA (F_ST_ = 0.565) involved in early embryogenesis and the gene TERF2IP/RAP1 (F_ST_ = 0.532) associated with the shelterin complex. A total of 12 GO terms were statistically overrepresented in the F_ST_ block on KEL6 and one on KEL17 (Table S7). Together, these results suggest that chromosome rearrangements may be playing a role in genomic differentiation between waterbuck karyotypes through supressed recombination on Rb chromosomes, with potential impacts for genic regions.

## 4 Discussion

### 4.1 Fine Scale Population Structure and Gene Flow Using Museum Collections

In this work we made use of available historical museum samples for the waterbuck in order to sample across a wider distribution of the species range and uncover finer‐scale population structure and gene flow across the species' distribution, than previously had been studied (Wang et al. 2024). Historical collections are becoming increasingly utilised for genomic studies, with the development of DNA extraction protocols and improvements in sequencing technologies (Raxworthy and Smith 2021). Our 24 historical samples grouped with the modern samples by geographic locality and heterozygosity at transversion sites was similar, suggesting that population structure has been maintained over the past 100 years. However, we found that genomic diversity at transition sites was higher in the historical samples, potentially due to minor DNA damage.

Through our combined historical and modern genomic dataset, our study emphasises the reduced gene flow across the Great Rift Valley and the Congolian Rainforest between population groups and subspecies than has previously been reported (Wang et al. 2024). This provides a more complete overview of the geographical barriers to gene flow in the waterbuck. Waterbuck have previously been reported to hybridise near the contact zone in Kenya and Tanzania (Lorenzen et al. 2006; Wang et al. 2024), and whilst our study also supports this admixture, we surprisingly find additional admixture between the two subspecies further from the contact zone across all samples within the Defassa South group.

These populations of defassa waterbuck in southern Africa represent an interesting group. Our increased sampling in Angola and DRC show that all individuals group between the two subspecies on the PC1 of the PCA and all have admixture proportions > 0.25 with common waterbuck. Based on a previous TreeMix analysis which showed a migration event from the common waterbuck population of Luangwa into the defassa population of Kafue (Wang et al. 2024), and our findings of admixture in both Luangwa and the southern defassa group, we suggest that gene flow may be bidirectional in this region. Our results also point to a barrier to gene flow between Matetsi (Common South) and the Defassa South group, with the Zambezi River potentially acting as a geographic barrier between the two populations.

These results reinforce the current taxonomic distinction between the two subspecies with some ongoing gene flow and support a broader north–south population structure across the species' range. They also underscore the critical role of museum collections in enabling robust inferences about population structure and gene flow—particularly in species with broad distributions and where genomic sampling may be difficult.

### 4.2 Genomic Differentiation Between Subspecies

Thanks to our new long‐read genome assembly, we were also able to study genomic differences between the two subspecies at the chromosome‐level. In the windows with the highest F_ST_ values we found the gene MTMR14, encoding for a myotubularin related protein that plays a role in autophagy (Gibbs et al. 2010), as well as the gene RTEL1, encoding a DNA helicase that maintains the integrity of telomeres and the stability of the genome by dissembling DNA secondary structures, facilitating DNA replication, repair and recombination (Vannier et al. 2014). Knocked‐out mice for this gene have been shown to have reduced cell proliferation, chromosomal fusions and heterogeneity of telomere lengths (Ding et al. 2004). Given the role of RTEL1 in maintaining telomere homeostasis and genome integrity, and the importance of telomeres in the formation of Rb fusions, it may therefore play a role in the polymorphic chromosome fusions in waterbuck. We also found the gene KATNAL1 which is involved in spermiogenesis, regulating chromosome alignment, segregation and cytokinesis during male meiosis. Its loss of function causes the disruption of microtubules, leading to male infertility (Smith et al. 2012). These results provide an overview of the potential impacts of genomic differentiation between subspecies on genes and a basis for further work to explore mechanisms that may be leading to speciation within this species.

### 4.3 Suppression of Recombination in Rb Fusions

Waterbuck exhibit karyotypic variation shaped by Robertsonian fusions (Kingswood et al. 1998; Kingswood et al. 2000). Using our chromosome‐level genome, we explored this in further detail, studying genomic differentiation and recombination patterns associated with both fixed (KEL3) and polymorphic (KEL6;17) Rb fusions.

Elevated F_ST_ and LD surrounding the centromere of KEL3—a fixed Rb fusion in *Kobus*—suggest reduced recombination in the pericentromeric region, consistent with linked selection, reduced diversity, and increased genomic differentiation in these regions. Rb fusions, whether in homozygous or heterozygous configurations, are known to alter the recombination landscape in pericentromeric regions, resulting in a redistribution of recombination towards interstitial and terminal chromosomal regions (Capilla et al. 2014; Dumas and Britton‐Davidian 2002; Marín‐García et al. 2024; Merico et al. 2013). While this may reflect an historical signature of the Rb fusion; centromeric effects alone cannot be excluded (Nambiar and Smith 2016; Talbert and Henikoff 2010). Alternatively, these results could also be indicative of a novel chromosome rearrangement, with similar indirect methods used in the seaweed fly to detect signatures of putative chromosome inversions (Mérot et al. 2021).

Regardless of the mechanism by which reduced recombination in the pericentromeric region of KEL3 occurred, the region on the p‐arm of KEL3 contained genes involved in male fertility, including the gene IFT140 which is associated with ciliated cells such as sperm (Zhang et al. 2018), PRSS21 involved in proteolytic events in the maturation of testicular germ cells (Netzel‐Arnett et al. 2009), and SEP12 which is involved in the elongation of sperm tails and the morphogenesis of sperm heads (Shen et al. 2017). The region of high F_ST_ on the q‐arm contained 15 genes and included OCA2, previously suggested to be involved in the variation in fur colouration within the species (Wang et al. 2024), as deletions within this gene can lead to albinism in cavefish (Protas et al. 2006).

For the polymorphic Rb fusion involving KEL6 and KEL17, we suggest that the homozygous Rb fusion of KEL6;17 in the common subspecies might have resulted in a region of high genomic differentiation and suppressed recombination surrounding the centromere, which may also be impacting regions across the Rb fused chromosome, in line with previous observations in mice (Marín‐García et al. 2024; Vara et al. 2021). We also find local population structure within these two regions using PCA. Following previous publications (e.g., Mérot et al. 2021; Wellband et al. 2019), local PCA clusters could reflect the three haplotypes of the Rb fusion (wildtype, heterozygous and homozygous), however this would suggest that the common subspecies is also polymorphic for the KEL6;17 fusion, having the heterozygous fusion. This has not been documented in previous cytogenetic studies (Kingswood et al. 1998; Pagacova et al. 2011). However, these previous studies focused predominantly on captive samples, whereas our study involves wild samples from across most of the waterbuck's distribution and therefore these karyotypes may have previously been unsampled. Further captive sampling of waterbuck karyotypes found evidence of another Rb fusion in the defassa subspecies (BTA7;29) (Pagacova et al. 2011), suggesting that other combinations of Rb fusions are possible in this species.

The high F_ST_ regions on KEL6 and KEL17 included several genes of functional interest. Among them, APELA which is implicated in early embryogenesis (Chng et al. 2013; Norris et al. 2017; Pauli et al. 2014) and the gene TERF2IP/RAP1 which protects telomeres from DNA damage and fusion events (De Lange 2005). Telomere loss or inactivation is required for the formation of Rb fusions (Slijepcevic 1998) and has previously been proposed in wild populations of house mice (Sánchez‐Guillén et al. 2015). This suggests that TERF2IP may play a role in facilitating the formation of Robertsonian fusions. Chromosome fusions may also result in the local adaptation of populations within a species, as described in Atlantic salmon and three‐spined stickleback (Liu et al. 2022; Wellband et al. 2019). This raises the possibility that the KEL6;17 fusion in waterbuck may similarly influence population‐level genomic divergence.

In contrast, we did not find similar genomic signatures of the Rb fusion on the other polymorphic chromosomes (KEL8 and KEL9). This could be because the high F_ST_ in the pericentromeric region in the polymorphic Rb fusion KEL6;17 is not a hallmark of the Rb, or more likely because KEL8;9 is only present in the 2*n* = 51 (heterozygous) and 2*n* = 50 (homozygous) karyotypes in the common subspecies (Kingswood et al. 1998), and these are either less frequent in wild populations and/or under sampled in our genomic study, limiting our ability to detect genomic differentiation within these regions.

Together, these results provide support for the recombination suppression model of chromosome evolution (Farré et al. 2013; Rieseberg 2001) and is consistent with patterns observed in other mammals such as mice (Capilla et al. 2014; Marín‐García et al. 2024; Vara et al. 2021). We identified regions of high LD surrounding the centromeres of some chromosomes involved in fixed and polymorphic Rb fusions, indicating reduced recombination within these areas. This has led to high levels of genomic differentiation and local population structure, potentially linked to the different karyotypes. Moreover, we find genes within these regions that are involved in fertility, development and recombination, key processes in reproductive isolation. However, the role of Rbs in conferring reproductive isolation in waterbuck is still debatable. We found admixed individuals in the hybrid zone with potentially heterozygous karyotypes, suggesting that if any, these Rbs might not provide a strong genetic barrier.

Karyotyping the animals used in this study is needed to further elucidate the role of Rbs in waterbuck evolution, with promise of techniques such as Hi‐C now able to screen frozen samples for chromosome rearrangements (Burden et al. 2025). The species is therefore an interesting model to further explore intraspecies chromosome evolution.

## 5 Conclusion

Our study provides a genome‐wide perspective on the evolutionary history of the African antelope, the waterbuck ( *Kobus ellipsiprymnus* ), which spans a broad geographic range across Africa. Utilising historical samples from museum collections, we expanded population coverage to previously unsampled regions, providing new insights into how climatic changes and geographic features have shaped population structure and gene flow between populations. Waterbucks' evolution is further complicated by karyotypic differences within and between the two subspecies due to Rb fusions. Here, we present evidence that Rb fusions can alter the recombination landscape, most notably on the fused chromosome KEL6;17 (BTA6;18), where suppressed recombination near centromeres is associated with elevated genomic differentiation. These findings suggest that Rb fusions may contribute to population divergence, although further genomic data (such as Hi‐C and long‐read sequencing) is needed to uncover the potential impacts of Rb fusions on both population structure and speciation.

## Author Contributions

Methodology, C.K., X.W., C.C.‐R., L.A.‐G., D.W., A.M., J.S.L., O.D., E.L.A., A.R.‐H., R.H., T.K. and M.F.; formal analysis, C.K., X.W., C.C.‐R., L.A.‐G. and D.W.; writing – original draft, C.K. and M.F.; writing – review and editing, C.K., C.C.‐R., O.D., A.R.‐H., R.H., T.K. and M.F.; supervision, M.F.

## Funding

This work was supported by Royal Society, RGS\R1\211047; Ministerio de Ciencia y Tecnología, Govern de Catalunya. PID2020‐112557GB‐I00; Agència de Gestió d'Ajuts Universitaris i de Recerca, 2021 SGR 0122; Institució Catalana de Recerca i Estudis Avançats; Ministerio de Economía y Competitividad, PRE‐2018‐083257; Behavioral Plasticity Research Institute, NSF DBI‐2021795; NSF Physics Frontiers Center, NSF PHY‐2210291; National Institutes of Health, RM1HG011016‐01A1.

## Ethics Statement

All the research complies with applicable laws on sampling from natural populations and animal experimentation (including the ARRIVE guidelines).

## Conflicts of Interest

The authors declare no conflicts of interest.

## Supporting information


**Figures S1–S13:** mec70218‐sup‐0001‐Figures.docx.

**Table S1:** Genome assembly curation.

**Table S2:** Gene annotation in the Defassa waterbuck genome.

**Table S3:** Percentage of transponsable elements in the Defassa waterbuck genome.

**Table S4:** Metadata of samples used in this study.

**Table S5:** Proportion of SNPs after filtering steps.

**Table S6:** List of genes in windows with high FSt.

**Table S7:** Gene Ontology enrichment of genes in windows with high FSt.

## Data Availability

The chromosome‐level genome prior to reordering and reorientation of chromosomes can be found at DNA Zoo (https://www.dnazoo.org/assemblies/kobus_ellipsiprymnus) and NCBI SRA (BioProject PRJNA512907). The historical WGS data can be found at NCBI SRA (BioProject PRJNA1232813). Custom code used as part of this project can be found at https://www.github.com/Farre‐lab/Kirkland_Waterbuck.
