# Supplementary material for: Chromosome‐Level Genomics and Historical Museum Collections Reveal New Insights Into the Population Structure and Chromosome Evolution of Waterbuck
Source: Mol Ecol. 2025 Dec 22;35(1):e70218. doi: 10.1111/mec.70218 (PMC12745853; doi:10.1111/mec.70218)
Supplement: Supplementary file 1 — Figures S1–S13: mec70218‐sup‐0001‐Figures.docx. [file MEC-35-e70218-s002.docx]

**Chromosome-level genomics and historical museum collections reveal new insights into the population structure and chromosome evolution of waterbuck**

Corey Kirkland^1^, Xi Wang^2^, Carla Canedo-Ribeiro^1^, Lucía Álvarez-González^3,4^, David Weisz^5^, Alexandria Mena^6^, Judy St Leger^7^, Olga Dudchenko^5,8^, Erez Lieberman Aiden^5,8^, Aurora Ruiz-Herrera^3,4^, Rasmus Heller^2^, Tony King^9,10^, Marta Farré^1*^

1. School of Natural Sciences, University of Kent, Canterbury, UK.
2. Department of Biology, University of Copenhagen, Copenhagen, Denmark.
3. Departament de Biologia Cellular, Fisiologia i Immunologia, Universitat Autònoma de Barcelona, Cerdanyola del Vallès, Spain.
4. Institut de Biotecnologia i Biomedicina, Universitat Autònoma de Barcelona, Cerdanyola del Vallès, Spain.
5. The Center for Genome Architecture, Baylor College of Medicine, Houston, USA.
6. SeaWorld San Diego, San Diego, CA, USA.
7. Cornell University College of Veterinary Medicine, Ithaca, NY, USA.
8. The Center for Theoretical Biological Physics, Rice University, Houston, USA.
9. The Aspinall Foundation, Port Lympne Reserve, Kent, UK.
10. Durrell Institute of Conservation and Ecology (DICE), University of Kent, Canterbury, UK.

* Correspondence: [m.farre-belmonte@kent.ac.uk](mailto:m.farre-belmonte@kent.ac.uk) (MF)

**Supplementary Materials**


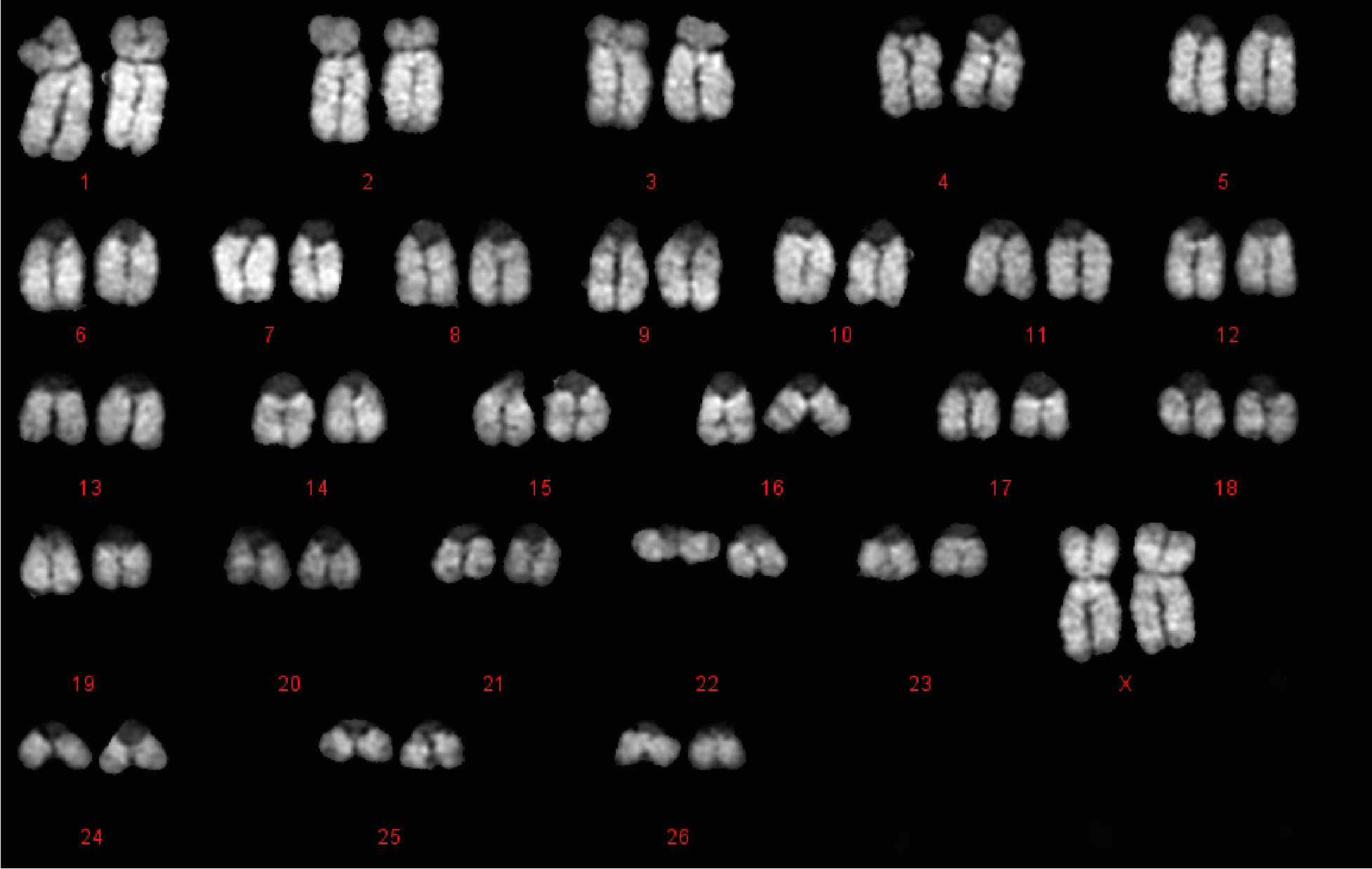


**Figure S1:** Defassa waterbuck cell culture karyotype (2*n* = 54, XX) stained with DAPI, with chromosomes named by size and the X chromosomes labelled separately.


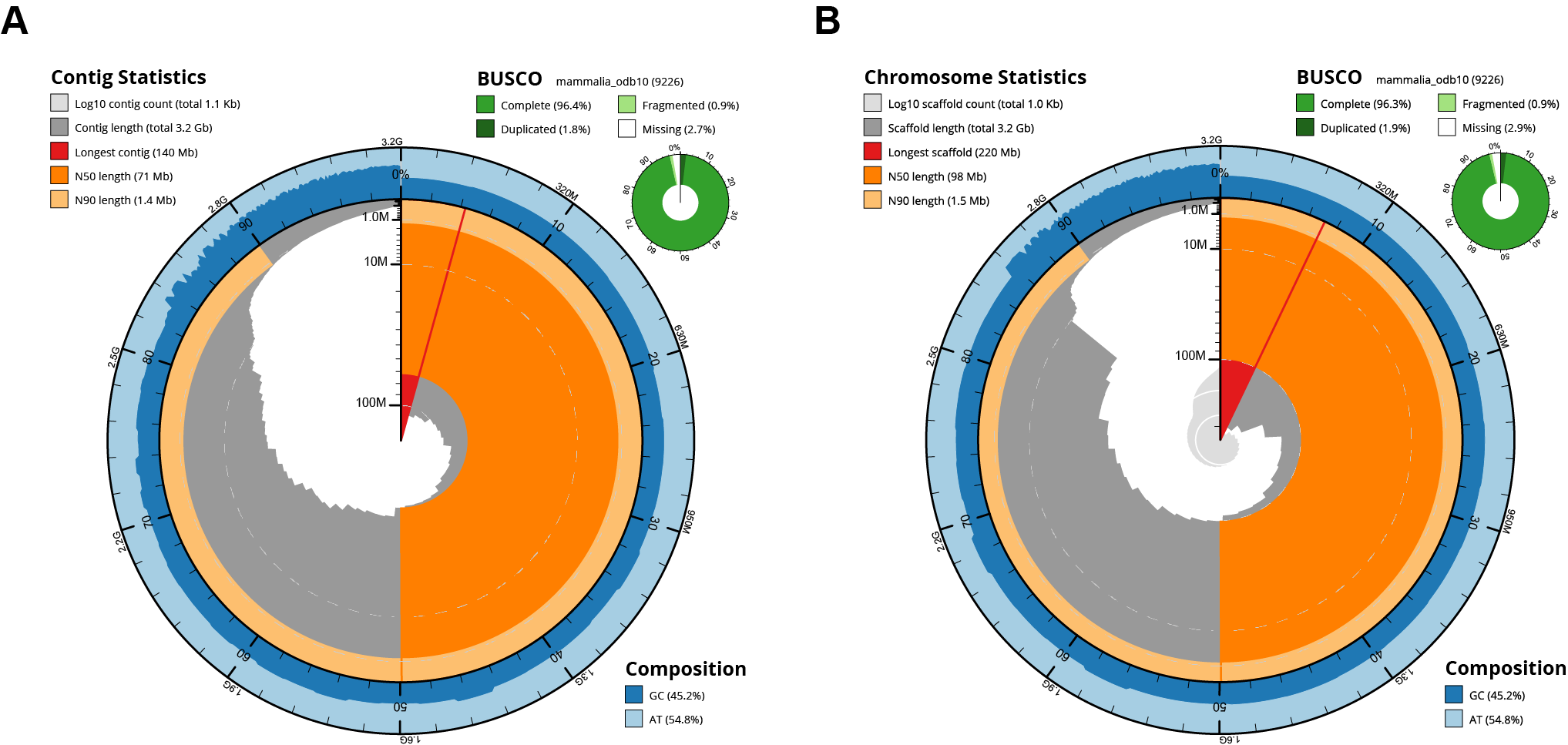


**Figure S2:** Snail plot of the Defassa waterbuck (A) contig-level and (B) chromosome-level genome assemblies.


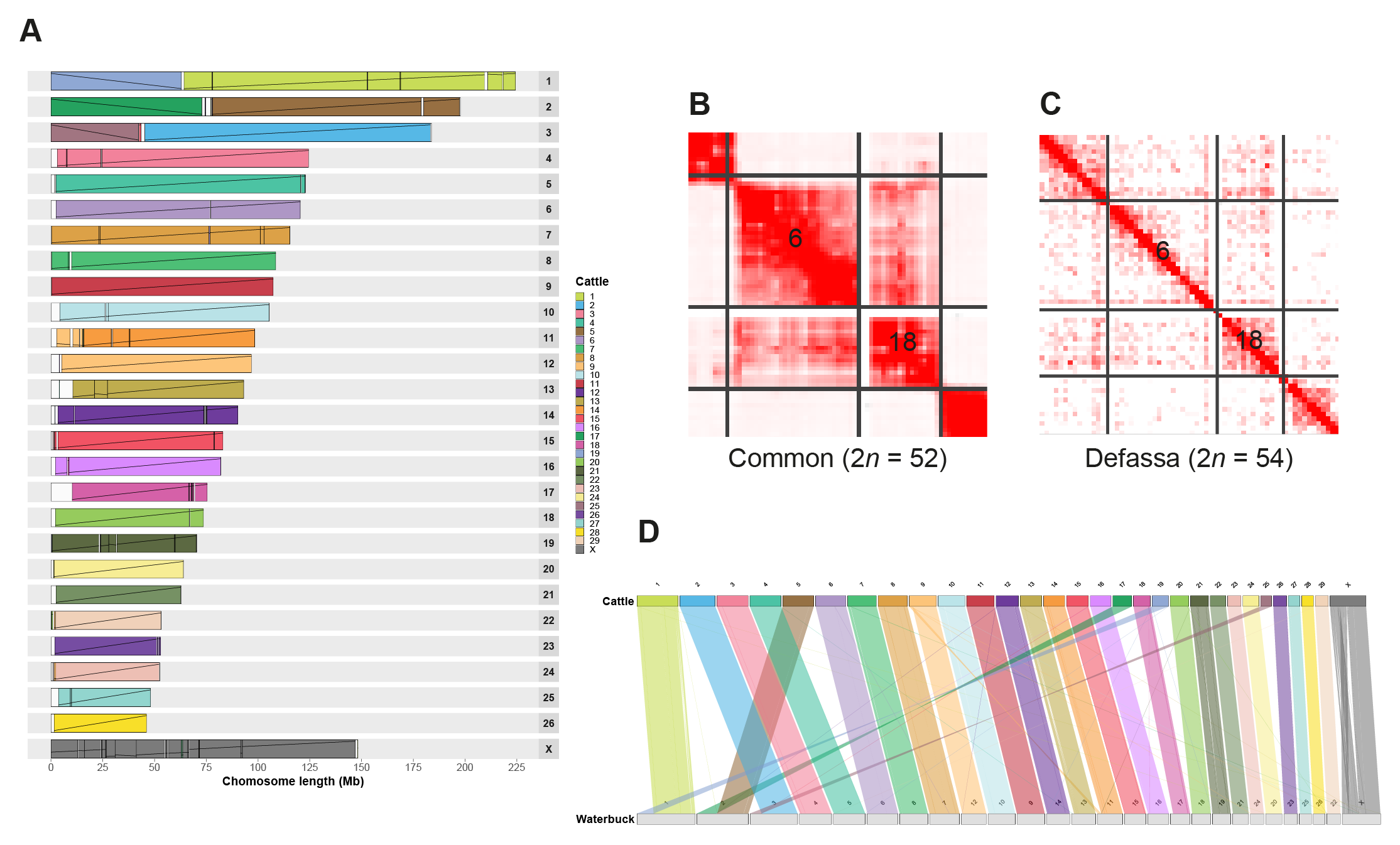


**Figure S3:** Waterbuck chromosome-level assembly after final genome curation. (A) Chromosome painting plot of the synteny between waterbuck and cattle genomes, with colours representing homology to cattle chromosomes. (B) Hi-C interaction matrix of the Hi-C sample 2*n* = 52, showing interactions between KEL6 and KEL17 (homologous to cattle BTA6 and BTA18, respectively) and (C) Hi-C sample 2*n* = 54. (D) Linear plot of the synteny between waterbuck and cattle chromosomes.


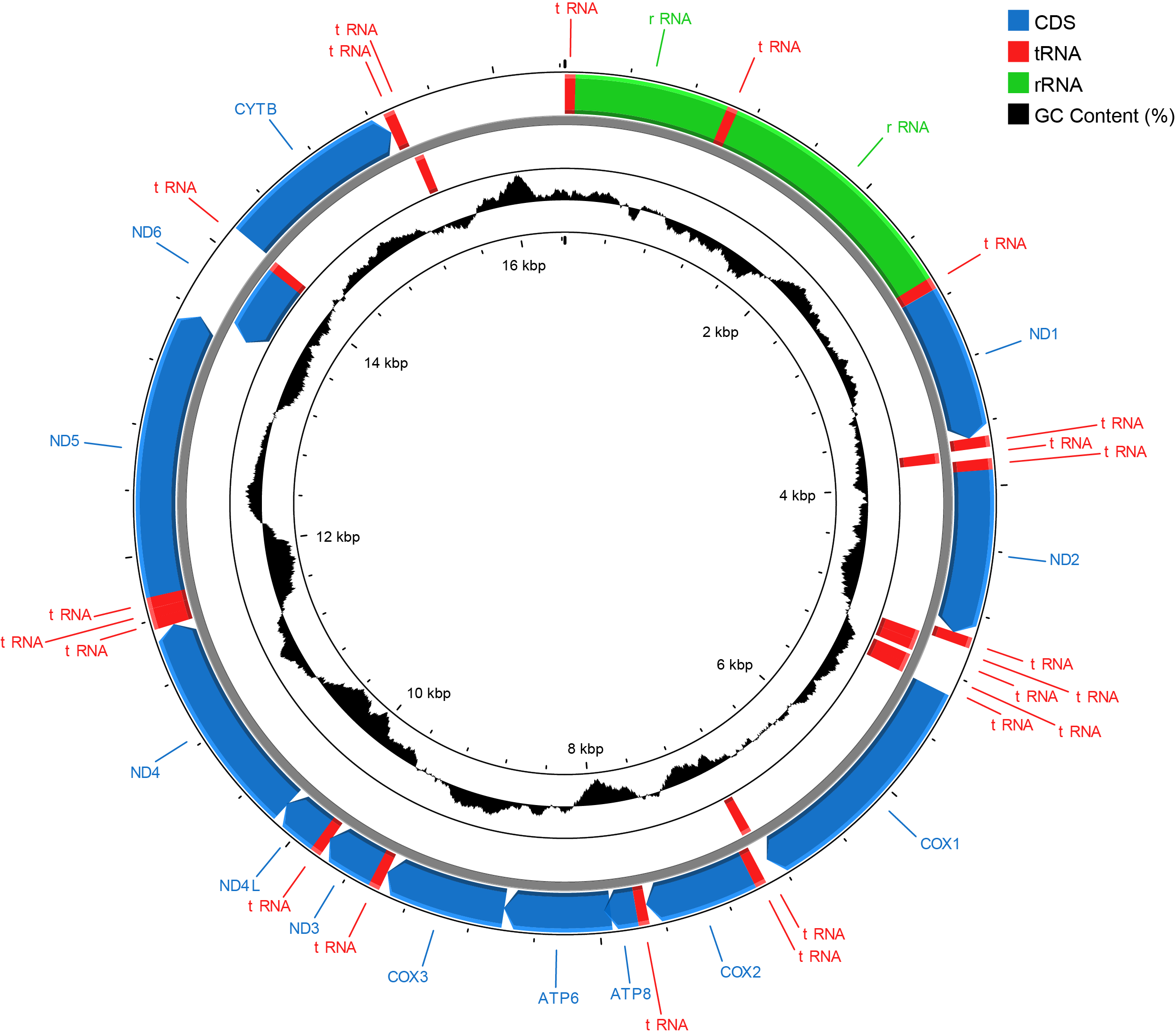


**Figure S4:** Mitochondrial genome assembled from the Defassa waterbuck PacBio HiFi reads, with coding sequences (CDS), tRNAs, rRNAs, and GC content (%) annotated.


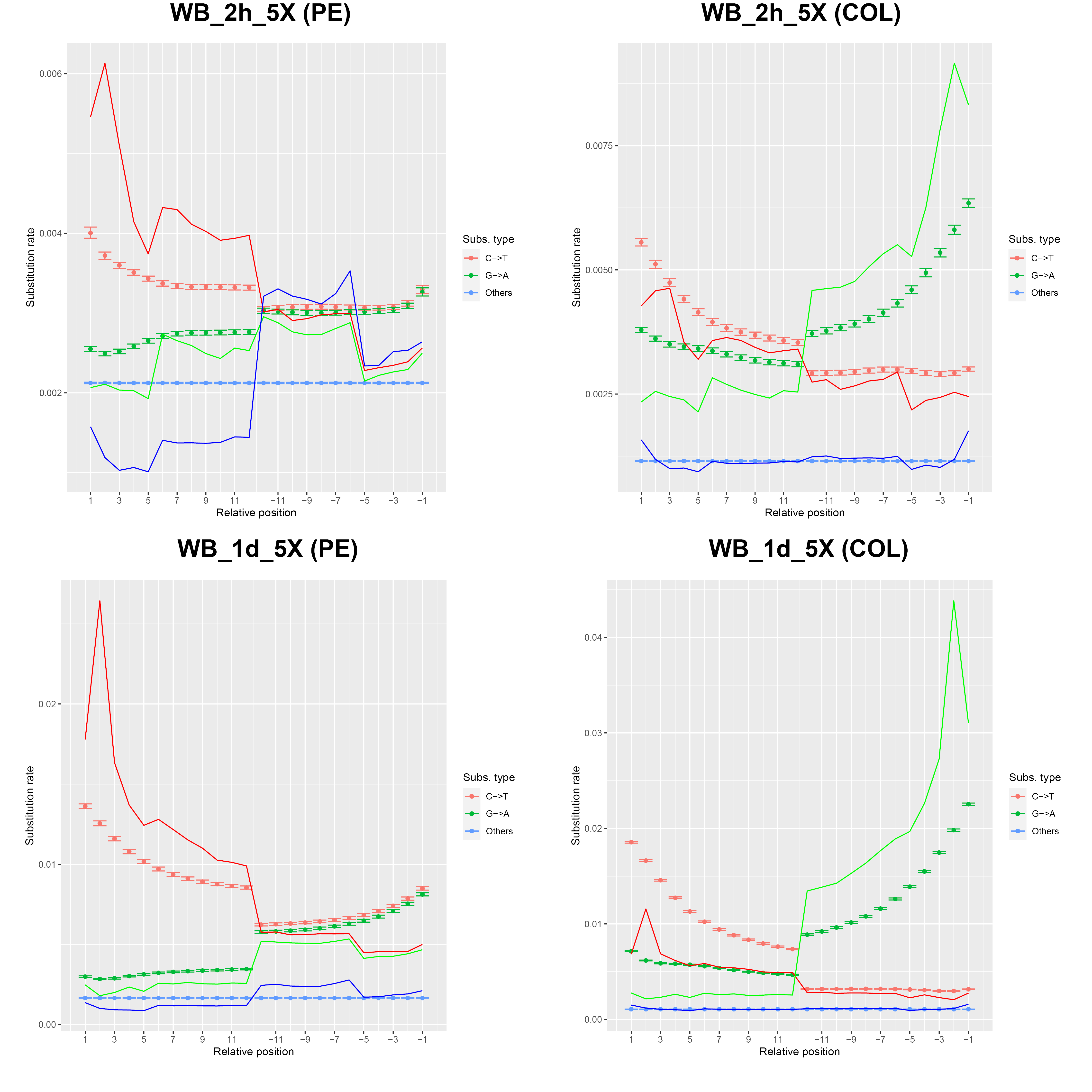


**Figure S5:** Estimation of DNA damage in the historical samples. Two of the samples with the highest (WB_1d_5X) and lowest (WB_2h_5X) substitution rates are shown. Both paired-end (PE) and collapsed (COL) mapped reads were analysed for each sample. Relative position refers to the position at the start and end of the read. Empirical misincorporation frequencies are denoted by the soil line. 95% simulated posterior predictive intervals of the fitted model are shown by the confidence intervals.


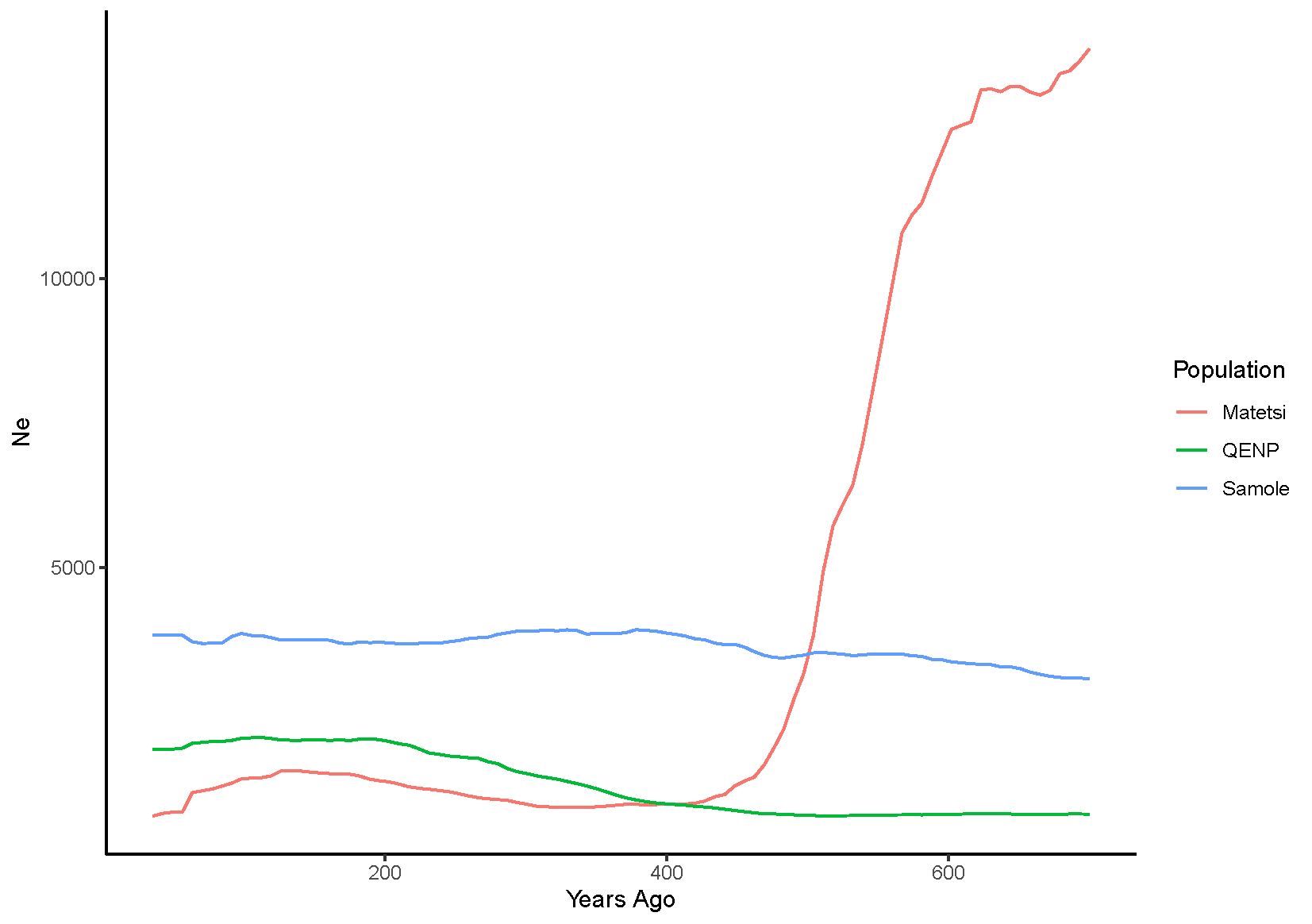


**Figure S6:** Effective population size (Ne) calculated using GONE2 for two defassa (Samole and QENP) and one common (Matetsi) waterbuck populations over the last 700 years (calculated with a generation time of 7).


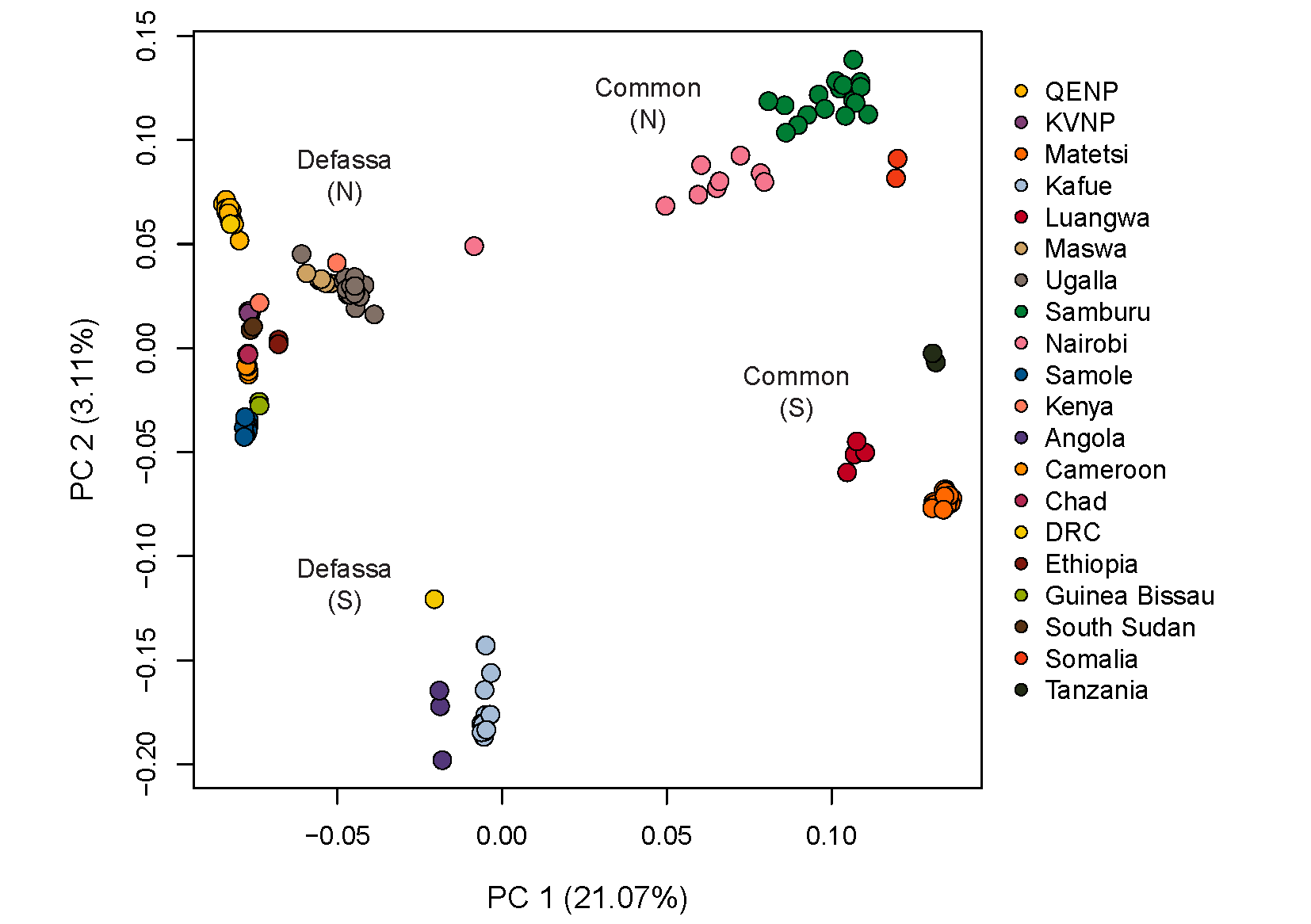


**Figure S7:** PCA of the 143 waterbuck samples using transversion genomic sites and coloured by population.


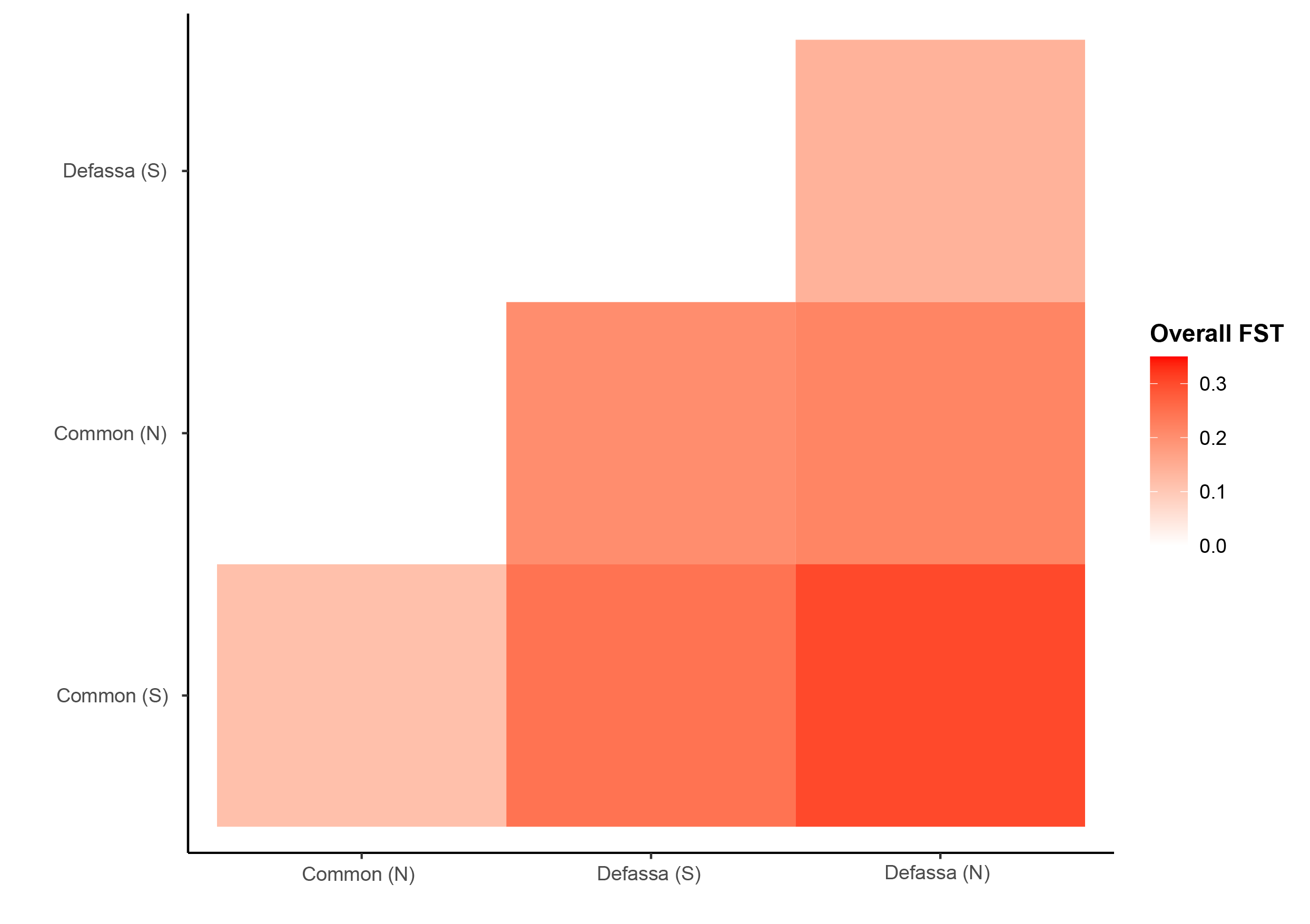


**Figure S8:** Overall F_ST_ between the waterbuck groups.


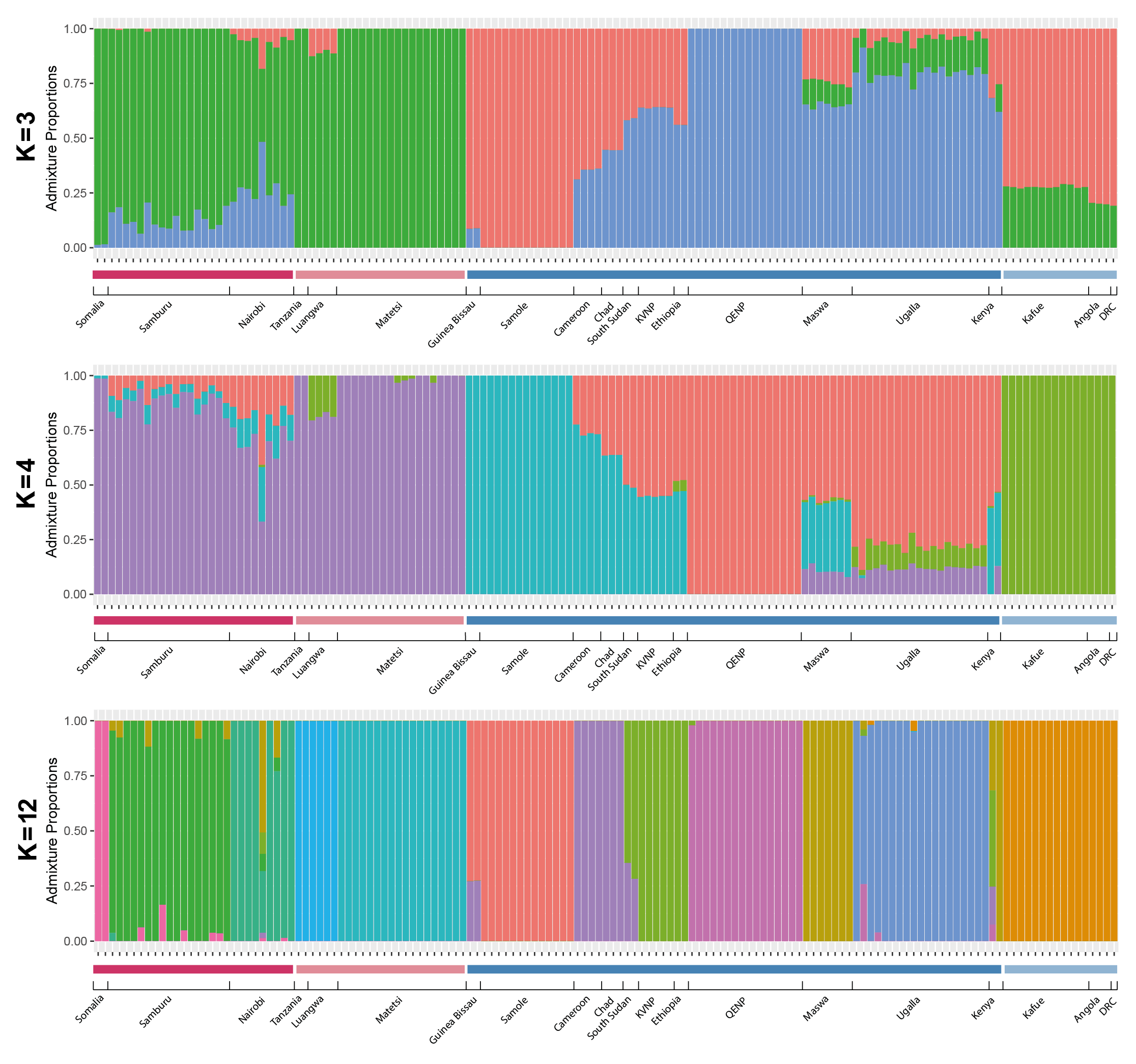


**Figure S9:** Admixture proportions of the waterbuck samples at K = 3, K = 4, and K = 12 estimated populations. Dark red is common (N), light red is common (S), dark blue is Defassa (N), and light blue is Defassa (S).


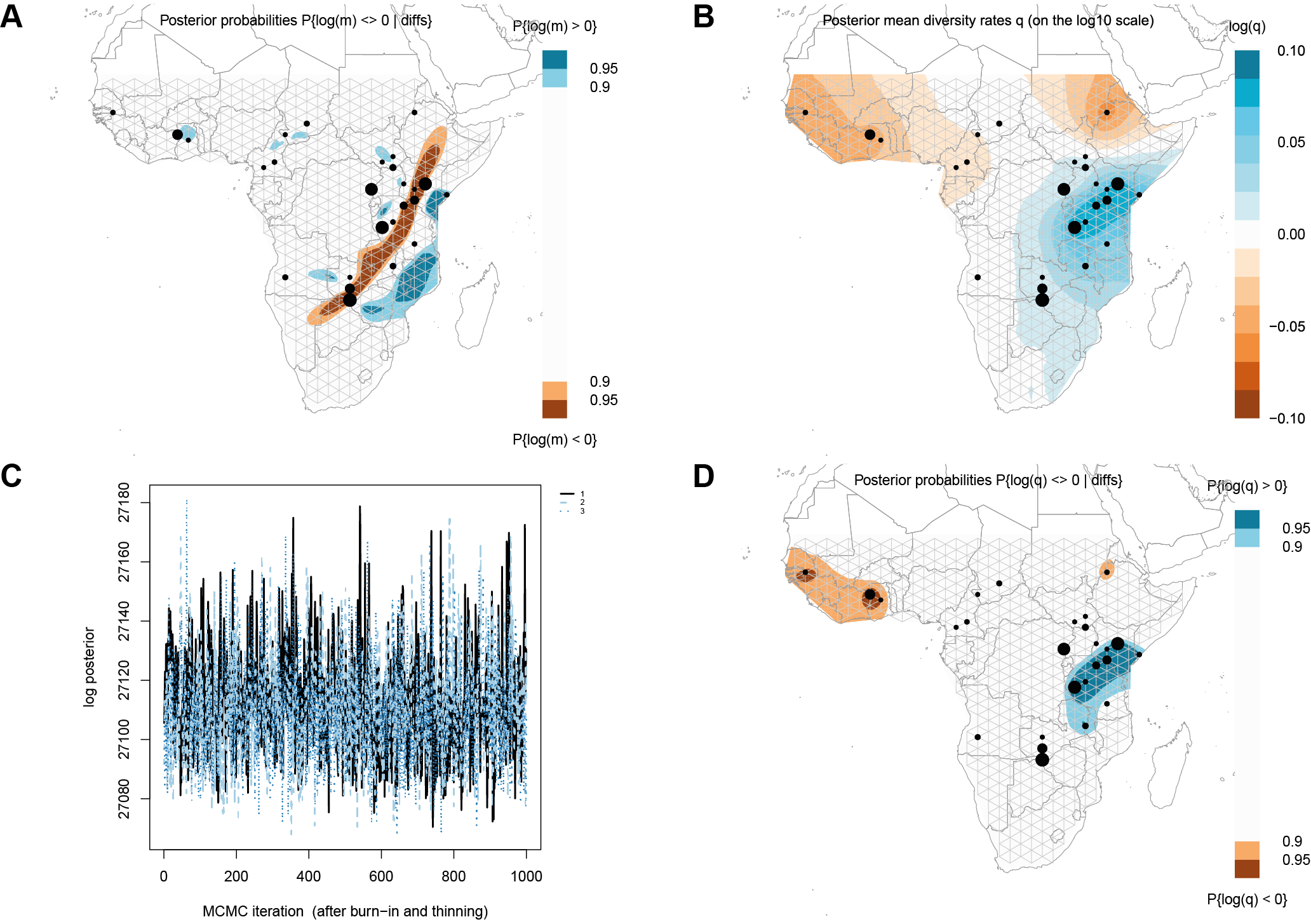


**Figure S10:** EEMS analysis of 142 waterbuck samples, excluding “WB_3k_5X”. (A) Maximum posterior probabilities of the migration rate (*m*). (B) Posterior mean diversity rates (*q*). (C) MCMC iterations showing convergence. (D) Maximum posterior probabilities of the diversity rate. Populations denoted by black circles, with size proportional to the number of individuals in each population.


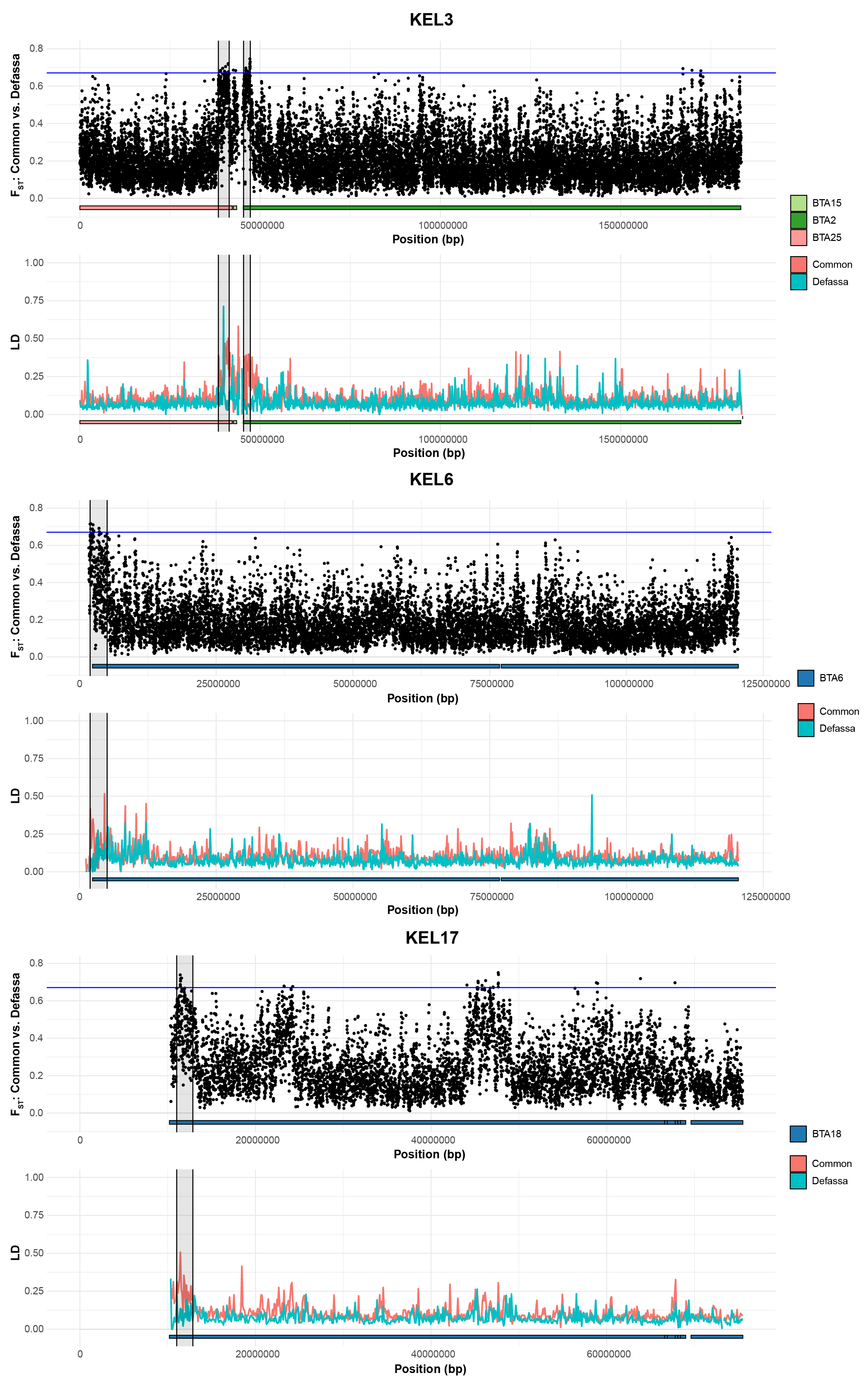


**Figure S11:** F_ST_ between waterbuck subspecies and mean LD in 100 Kb windows for each subspecies across chromosomes KEL3, KEL6, and KEL17 with fixed or polymorphic Rb fusions. Homology to cattle chromosomes (BTA) shown. Regions of interest highlighted.


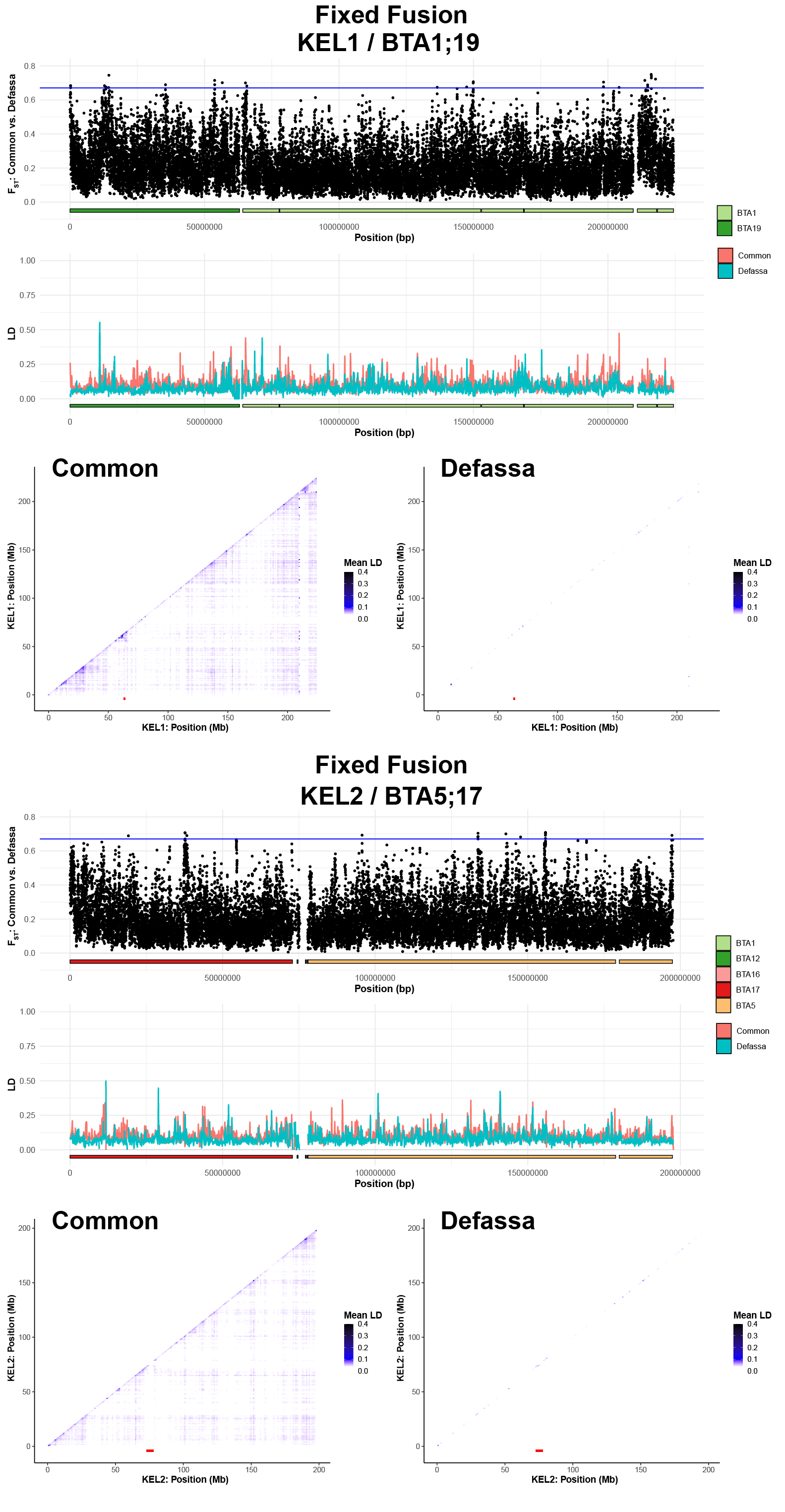


**Figure S12:** F_ST_ between subspecies, mean LD in 100 Kb windows for each subspecies, and mean pairwise LD in 1 Mb windows for each subspecies across chromosomes KEL1 and KEL2 involved in fixed Rb fusions in waterbuck. Homology to cattle chromosomes (BTA) shown. Red line shows the putative centromeric region.


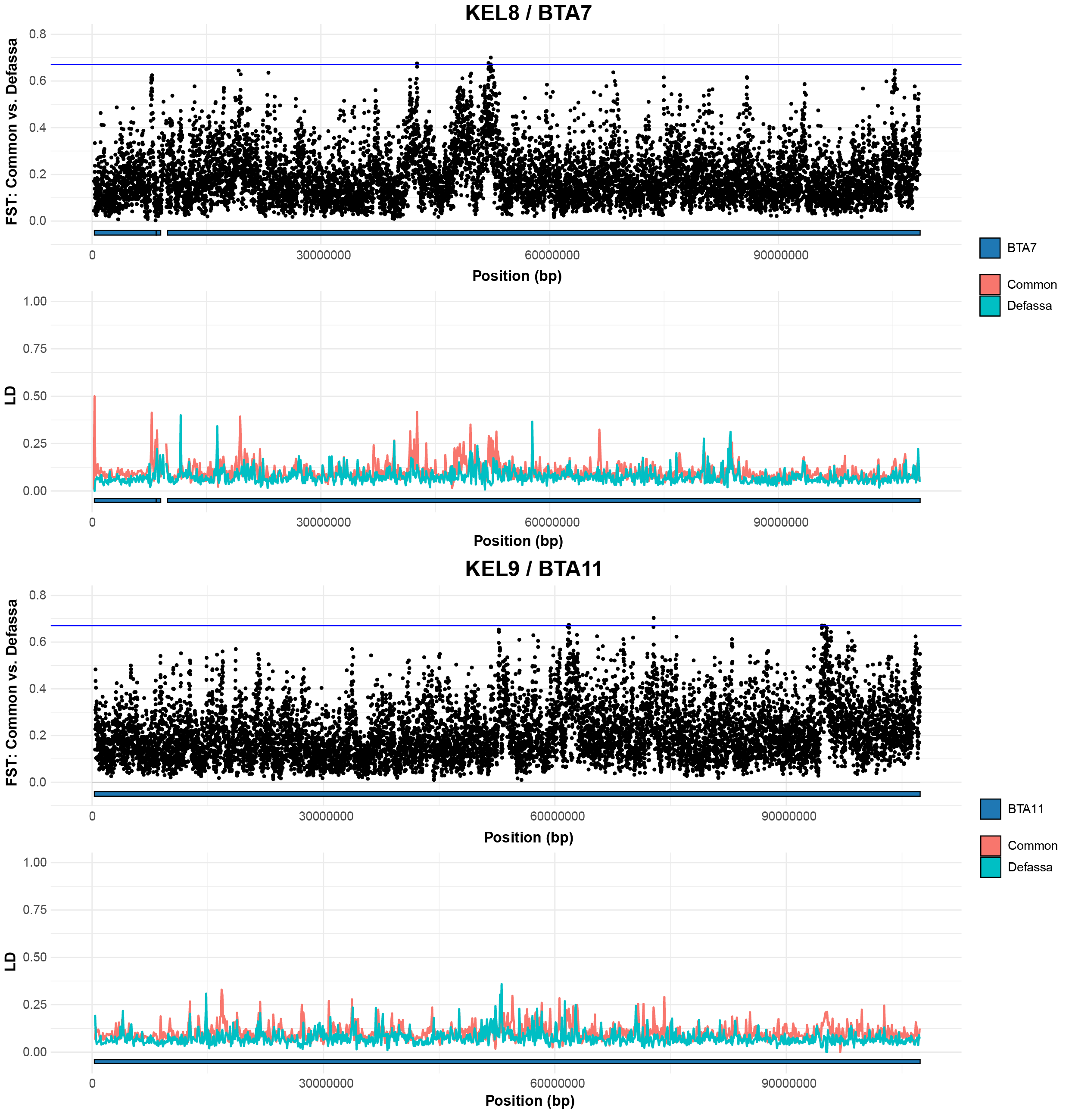


**Figure S13:** F_ST_ between waterbuck subspecies and mean LD in 100 Kb windows for each subspecies across chromosomes KEL8 and KEL9 involved in the polymorphic Rb fusion. Homology to cattle chromosomes (BTA) shown.
